# Immune and Tumor Microenvironment Mechanisms of *Hedyotis diffusa* Willd: A Scoping Review and Network Pharmacology Analysis

**DOI:** 10.3390/cancers18040672

**Published:** 2026-02-18

**Authors:** Soo-Dam Kim, Eun Soo Park, Jung Hyang Park, Tae-Kyung Yeo, Dong-Hyeon Kim, So-Jung Park, Hwa-Seung Yoo

**Affiliations:** 1 KM Science Research Division, Korea Institute of Oriental Medicine, Daejeon 34054, Republic of Korea; hidden425@kiom.re.kr; 2 East West Cancer Center, Daejeon Korean Medicine Hospital, Daejeon University, Daejeon 34520, Republic of Korea; jses09514@naver.com (E.S.P.); wjdgidqkr@naver.com (J.H.P.); 3 Oculi Korean Medicine Hospital, Seoul 05655, Republic of Korea; oculi2022@naver.com; 4 Korean Medicine Hospital, Pusan National University, Yangsan 50612, Republic of Korea; dongxian92@gmail.com; 5 Department of Korean Internal Medicine, School of Korean Medicine, Pusan National University, Yangsan 50612, Republic of Korea

**Keywords:** *Hedyotis diffusa* Willd, tumor microenvironment, immunomodulation, network pharmacology, herbal medicine

## Abstract

**Background:** The tumor microenvironment (TME) is a dynamic ecosystem that critically shapes tumor progression, immune escape, and therapeutic responses. *Hedyotis diffusa* Willd (HDW) has long been used in East Asian medicine for conditions associated with inflammation and malignancy, yet its immunological and microenvironmental mechanisms have not been systematically synthesized. **Methods:** A scoping review of preclinical studies (2016–2025) was conducted following the Arksey-O’Malley framework and PRISMA-ScR guidance to systematically map experimental evidence on the antitumor, immune-related, and TME-associated effects of HDW. To complement and interpret these heterogeneous experimental findings at a systems level, a network pharmacology analysis was performed to identify bioactive compounds, predict their putative protein targets, and assess their convergence on immune- and microenvironment-related signaling networks through protein-protein interaction and pathway enrichment analyses. **Results:** Fifty-nine studies met eligibility criteria, encompassing in vitro and in vivo models across diverse cancer types. Experimental evidence from preclinical models showed that HDW extracts and bioactive fractions consistently suppressed proliferation, induced apoptosis and ferroptosis, inhibited epithelial-mesenchymal transition (EMT) and angiogenesis, and enhanced cytotoxic lymphocyte activity, while attenuating tumor-associated inflammation. In parallel, in silico network analysis identified 94 intersecting immune- and TME-related targets and revealed a densely connected interaction network centered on PI3K-Akt, STAT3, EGFR, and SRC. Enrichment analyses highlighted receptor tyrosine kinase signaling, inflammatory pathways, metabolic regulation, and focal adhesion as dominant functional themes. **Conclusions:** HDW acts as a multi-target botanical agent that integrates direct cytotoxicity with immune activation and TME remodeling. Network-in-formed interpretation indicates that the modulation of PI3K-Akt-STAT3 signaling is a common mechanistic axis linking experimental observations.

## Simple Summary

Cancer growth is strongly influenced by its surrounding environment, which includes immune cells, blood vessels, and supporting tissues. Many traditional herbal medicines are used alongside cancer care, but their effects on this tumor environment are not always clearly understood. *Hedyotis diffusa* Willd (HDW) is a commonly used herb in East Asian medicine, yet its actions on immune responses and the tumor environment have not been comprehensively summarized. In this study, we reviewed existing laboratory research and combined it with computational network analysis to understand how this herb may work. We found that HDW can suppress cancer cell growth, reduce harmful inflammation, and support anticancer immune activity through multiple biological pathways. These findings provide an integrated picture of how this herbal medicine may influence cancer-related immune and microenvironmental processes, offering a useful reference for future experimental and translational research.

## 1. Introduction

The concept of the tumor microenvironment (TME) has become central to modern cancer biology. Tumors develop within a complex setting that includes malignant cells, immune cells, stromal components, extracellular matrix structures, and extensive vascular support [1,2]. Disturbances within this environment, such as persistent inflammation, im-paired immune surveillance, abnormal cytokine signaling, and uncontrolled angiogenesis, promote cancer progression and allow malignant cells to evade immune control [3,4,5]. As immuno-oncology advances, therapeutic strategies increasingly concentrate on strengthening antitumor immunity. Current approaches include restoring the activity of cytotoxic T-cells, redirecting the behavior of tumor-associated macrophages, and reducing immunosuppressive features within the microenvironment [6,7,8]. These developments have encouraged researchers to explore natural compounds that can influence multiple immune-related pathways at the same time, which has increased interest in botanical medicines with broad immunomodulatory potential [9,10].

*Hedyotis diffusa* Willd (HDW), a herb widely used in East Asian medical systems, has traditionally been applied for its detoxifying and anti-inflammatory purposes. Modern pharmacological investigations have expanded this view by showing that HDW demonstrates antitumor activity in various in vitro and in vivo models [11,12,13]. Chemical analyses have identified triterpenoids, flavonoids, iridoids, and anthraquinones as major groups of bioactive compounds that contribute to its pharmacological effects [14,15]. Experimental studies report that HDW reduces cancer cell proliferation, induces apoptosis, limits new blood vessel formation, and modulates inflammatory responses in several malignancies, including colorectal, liver, breast, gastric, lung, and hematologic cancers [16,17,18,19,20]. Despite these diverse findings, earlier discussions have usually focused on mechanisms relevant to specific cancer types. As a result, its potential influence on immune regulation, cytokine networks, macrophage activity, and broader microenvironmental processes has not been described in a fully integrated manner.

Network pharmacology studies have attempted to explain the mechanisms of HDW, yet most previous analyses concentrated on individual diseases such as colorectal cancer, hepatocellular carcinoma, or lung cancer [21,22,23,24]. Accordingly, prior reviews and network-based analyses have largely remained disease-centered, without explicitly synthesizing immune regulation or tumor microenvironment-related mechanisms across cancer types. This fragmented approach has limited a comprehensive understanding of the immunological relevance of HDW, despite accumulating preclinical evidence suggesting its immune- and microenvironment-modulatory potential [25,26,27]. More specifically, HDW-derived flavonoid fractions have been shown to suppress pro-inflammatory cytokine production, including IL-6 and TNF-α, in macrophage models through the inhibition of NF-κB-related signaling [25]. In addition, HDW polysaccharides have been reported to activate the IL-2/IL-2R axis and enhance antitumor immune responses in murine colorectal cancer models, improving immunotherapy-related outcomes and suggesting the attenuation of immune escape-related processes within the tumor microenvironment [27]. However, these findings have not been synthesized within a cross-disease framework that links experimental observations to immune- and TME-associated molecular targets.

To address this gap, we conducted a scoping review of preclinical studies to map the immune- and TME-related effects of HDW across diverse tumor models. We then performed a targeted network pharmacology analysis focused on immune- and TME-associated molecular targets and pathways to support a systems-level interpretation of the mapped evidence. This integrated approach aims to identify shared mechanistic nodes with translational relevance and to facilitate hypothesis generation for future biomarker- or immunotherapy-oriented evaluations of HDW.

## 2. Methods

### 2.1. Study Design

This study consisted of two complementary components. First, a scoping review was conducted to map preclinical evidence on the antitumor, immune- and TME-related effects of HDW. Second, a network pharmacology analysis was performed to explore how active compounds of the herb may interact with immune- and microenvironment-associated targets at a systems level. The scoping review provided an evidence map of experimentally reported immune- and TME-related effects of HDW, which was used to contextualize the network-derived targets and pathways and to frame the network pharmacology results as hypothesis-generating.

### 2.2. Scoping Review

The scoping review was conducted according to the methodological framework developed by Arksey and O’Malley, and the reporting process followed the PRISMA-ScR guideline [28,29]. The review protocol was registered with the Open Science Framework (OSF) on 12 October 2025 (https://osf.io/qjmpy). In addition, the Joanna Briggs Institute methodology for scoping reviews was consulted to supplement the methodological approach [30]. The objective of this review was to organize and classify existing preclinical findings related to the biological actions of HDW, including antitumor activity, immune-associated responses, and TME-related mechanisms. The review identified the types of cancer models used, the experimental designs applied, the outcomes measured, and the main findings reported across the studies.

#### 2.2.1. Search Strategy

A comprehensive literature search was carried out in PubMed, Embase, CENTRAL and OASIS. OASIS was included to capture Korea-indexed studies on HDW that may not be fully indexed in international databases, thereby improving coverage of East Asian evidence. The search included studies published from January 2016 to December 2025. Search terms consisted of controlled vocabulary and free-text words related to the herb and cancer research. The primary terms included “*Hedyotis diffusa*,” “Bai Hua She She Cao,” and cancer-related terms such as “cancer,” “tumor,” “carcinoma,” and “neoplasm.” Immune- and TME-specific keywords were not added to the core search to avoid excessive non-cancer retrieval and to preserve sensitivity, and immune/TME relevance was applied during eligibility assessment and data extraction. These terms were combined using Boolean operators appropriate to each database (Supplementary Materials Table S1). A representative full search string for PubMed was as follows: ((cancer*) OR (carcinoma*) OR (neoplasm*) OR (tumor*)) AND ((Hedyotis diffusa*) OR (Oldenlandia diffusa*) OR (Baihuasheshecao*)). Searches were performed without language restrictions at the retrieval stage. However, the final eligibility assessment was limited to studies with accessible full texts published in English.

#### 2.2.2. Eligibility Criteria

Studies were considered eligible if they met the following conditions: (1) preclinical experiments using in vitro or in vivo models of cancer; (2) the intervention consists of HDW extracts, isolated fractions, or identified active compounds from the herb; (3) outcomes include antitumor effects or biological responses relevant to immune or microenvironmental processes. Studies involving multi-herb formulas that did not allow the evaluation of the specific contribution of HDW were excluded. Reviews, commentaries, and conference abstracts without extractable data, as well as studies without accessible full text, were excluded. Clinical studies were excluded because this scoping review focused on preclinical models to synthesize mechanistic evidence. To ensure consistent interpretation and data extraction, the final inclusion was restricted to English-language full-text articles.

#### 2.2.3. Study Selection Process

All retrieved records were imported into a reference management program to remove duplicates. Two reviewers independently screened titles and abstracts to identify potentially eligible studies. Full texts of selected articles were then assessed for final inclusion. Disagreements were resolved through discussion or by consulting a third reviewer. The selection process was presented using a PRISMA-ScR flow diagram.

#### 2.2.4. Data Extraction and Synthesis

A standardized extraction form was used to collect information from the included studies. Extracted data included publication year, country, cancer type, experimental model, type of HDW preparation, dose and exposure conditions, measured outcomes, and main findings. Two reviewers independently extracted data, and discrepancies were resolved through discussion and consensus. If needed, a third reviewer adjudicated unresolved discrepancies. Data were organized to identify patterns across studies, such as frequently reported mechanisms, common signaling pathways, and recurring biological responses. Because this review was exploratory, findings were synthesized descriptively. Results were summarized according to cancer types, experimental models, types of HDW preparations, and major categories of biological effects.

### 2.3. Network Pharmacology Analysis

The network pharmacology analysis was conducted as a hypothesis-generating approach to explore how active compounds of HDW interacted with immune- and TME-related targets at a systems level, following principles of network-based drug discovery and traditional medicine systems pharmacology [31,32]. The analysis proceeded through four main steps: identification of candidate active compounds, prediction of potential human protein targets, collection of immune- and TME-related gene sets, and construction and interpretation of protein-protein interaction (PPI) networks and enriched pathways.

#### 2.3.1. Identification of Candidate Active Compounds

Candidate active compounds of HDW were identified using the Traditional Chinese Medicine Systems Pharmacology Database and Analysis Platform (TCMSP), accessed on 5 September 2025 [33]. All compounds registered for HDW were retrieved, and oral bio- availability (OB) and drug-likeness (DL) values were extracted. In line with previous systems pharmacology studies, compounds were retained as candidates if they satisfied the thresholds of OB ≥ 30% and DL ≥ 0.18.

#### 2.3.2. Target Prediction for Active Compounds

Potential human protein targets for each selected compound were predicted through SwissTargetPrediction [34]. The simplified molecular input line entry specification (SMILES) for each compound was obtained from TCMSP or from the PubChem database and was submitted to SwissTargetPrediction with the species parameter set to Homo sapiens. For each compound, predicted targets with probability ≥ 0.09 were extracted, and targets with probability < 0.09 were removed to reduce noise. All predicted targets were combined into a single list, and duplicate entries were removed based on gene symbol or UniProt identifier.

#### 2.3.3. Collection of Immune and TME-Related Gene Sets

Immune-related genes were collected from the Immunology Database and Analysis Portal (ImmPort), which provides curated gene lists associated with immune functions [35]. TME-related genes were identified from the GeneCards database using the search term “tumor microenvironment” [36]. Genes with a relevance score ≥ 2 were retained. The immune-related genes from ImmPort and the TME-related genes from GeneCards were merged into a single gene set after the removal of duplicate symbols. All databases were accessed on 5 September 2025.

#### 2.3.4. Identification of Intersecting Targets and PPI Network Construction

Intersecting targets between the predicted HDW-related targets and the immune and TME gene set were identified using spreadsheet software. The overlap was computed based on official gene symbols, and the resulting intersecting genes were considered as candidate mediators of immune- and microenvironment-related actions of HDW. These intersecting targets were then submitted to the STRING database (Search Tool for the Retrieval of Interacting Genes or Proteins, version 12.0) to construct a PPI network, with the organism set to Homo sapiens and a minimum required interaction score of 0.4 [37]. The interaction data were exported from STRING for subsequent network visualization and analysis.

#### 2.3.5. Network Visualization and Identification of Key Targets

The PPI network was imported into Cytoscape (version 3.10.4), an open source software platform for visualizing complex networks [38]. Topological properties such as degree and betweenness centrality were calculated using the network analysis functions within Cytoscape to identify highly connected or central nodes as potential key targets. Network clustering was performed with the MCODE plug-in to detect densely connected modules that might represent functional sub-networks [39]. Modules with relatively high scores and clear biological coherence were selected for further interpretation.

#### 2.3.6. Functional Enrichment Analysis

Functional enrichment analysis was performed to investigate the biological path-ways and processes associated with the intersecting targets and with key network modules. The Kyoto Encyclopedia of Genes and Genomes (KEGG) pathway and Gene Ontology biological process (GO-BP) enrichment were conducted using the enrichment tools provided within the STRING platform [40,41]. Multiple testing was corrected using the Benjamini-Hochberg false discovery rate (FDR) method, and terms with FDR-adjusted *p*-values < 0.05 were considered significant. Pathways and biological processes with statistically significant enrichment were examined, with a focus on immune signaling, cytokine and chemokine networks, leukocyte activation, macrophage polarization, and angiogenesis. The results of the network pharmacology analysis were integrated with findings from the scoping review to propose a coherent mechanistic framework for the actions of HDW in the context of antitumor immunity and the TME.

## 3. Results

### 3.1. Study Selection

**Figure 1:**
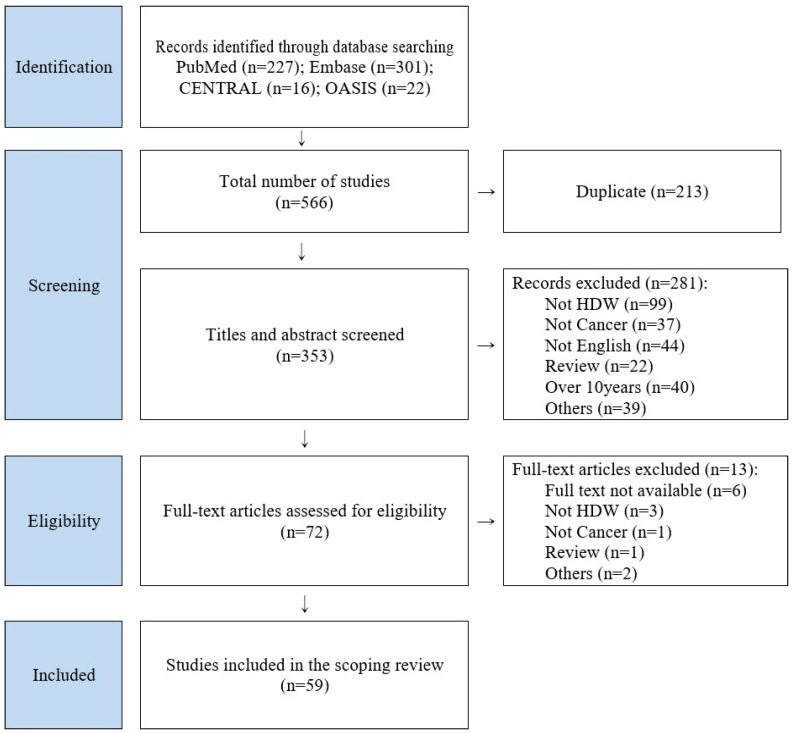
PRISMA study flow chart.

A total of 566 records were identified through database searching. After removing 213 duplicates, 353 studies remained for title and abstract screening. During this screening stage, 288 records were excluded for the following reasons: not related to HDW (*n* = 99), not cancer-related (*n* = 37), not published in English (*n* = 44), review articles (*n* = 22), published more than 10 years ago (*n* = 40), or excluded for other reasons (conference abstracts or protocol-only records) (*n* = 39). Following this process, 72 full-text articles were assessed for eligibility. Of these, 13 articles were excluded at the full-text stage for the following reasons: full text not available (*n* = 6), not HDW (*n* = 3), not cancer (*n* = 1), review (*n* = 1), and others (*n* = 2). Finally, 59 studies met the eligibility criteria and were included in the scoping review. A summary of the study selection process is presented in Figure 1.

### 3.2. Characteristics of Included Studies

Most of the eligible studies were conducted in China (*n* = 49), with a smaller number originating from other countries (*n* = 10). Across all included studies (*n* = 59), a majority of the included studies used in vitro experimental systems (*n* = 32), whereas others combined both in vitro and in vivo approaches (*n* = 24). Only a small number relied exclusively on animal models (*n* = 3). Across the studies, a wide range of cancer types was represented. The most frequently investigated models were colorectal cancer (16 studies), lung cancer, including NSCLC and adenocarcinoma (10 studies), and hepatocellular carcinoma (8 studies). Sample sizes varied substantially, particularly among animal studies, where treatment groups ranged from 4 to 15 animals. A summary of representative study characteristics is provided in Table 1.

### 3.3. Antitumor Activity and Growth Inhibition

#### 3.3.1. Inhibition of Cancer Cell Proliferation and Cell Cycle Arrest

Across diverse tumor models, HDW and its derivatives consistently suppressed cancer cell growth. Crude extracts, polysaccharide fractions, total flavonoids, and single constituents such as quercetin and ursolic acid reduced cell viability and/or colony formation in breast, colorectal, hepatocellular, lung, and bladder cancer, while showing limited toxicity in non-malignant lines [42,44,45,47,51,65,66,67,76,88]. Quercetin decreased MCF-7 viability together with the downregulation of BIRC5 and CDK1 [45], and HDW-derived exosome-like particles (HDW-EVLPs) inhibited Huh-7 growth with minimal impact on WRL68 hepatocytes [13]. HDW selectively reduced A549 cell viability but spared WI-38 fibroblasts, indicating a degree of tumor selectivity [65]. Many of these studies also reported cell cycle arrest at G0/G1, G1/S, or G2/M, accompanied by changes in cyclins, CDKs, and p21, suggesting a direct interruption of cell cycle progression and DNA replication [12,22,26,44,60,62,66,67,72,73,74,83,85,86].

#### 3.3.2. Induction of Apoptosis and Other Regulated Cell Death Pathways

Growth inhibition was closely linked to the activation of programmed cell death. Numerous HDW-based preparations triggered classical apoptosis characterized by chromatin condensation, Annexin V positivity, mitochondrial depolarization, caspase-3/8/9 activation, and PARP cleavage, together with a shift in Bcl-2 family proteins toward a proapoptotic profile (Bax/Bad upregulation with Bcl-2/Bcl-xL downregulation) [47,48,55,56,60,62,65,66,67,72,89,91]. ODE and ethanol extracts from HDW induced caspase-dependent mitochondrial apoptosis in colorectal cancer, with increased Bax, Apaf-1, and cytosolic cytochrome c [66,67]. Additional forms of regulated cell death were also identified. In bladder cancer, HDW decreased GPX4 and SLC7A11 and increased TFRC and HMOX1, together with tumor shrinkage, supporting ferroptosis-mediated antitumor effects [42]. Lung cancer models exposed to HDW-derived injections showed lipid ROS accumulation and Fe^2+^ overload consistent with ferroptotic damage [19]. Flavonoid-rich fractions and other preparations enhanced LC3B-II expression and autophagic flux while simultaneously reducing viability and tumor burden, indicating an autophagy-associated cytotoxic mechanism in selected systems [44,82]. Overall, HDW appears capable of engaging multiple, context-dependent regulated cell death pathways.

#### 3.3.3. Suppression of Tumor Growth and Systemic Safety in Animal Models

In vivo findings generally mirrored the in vitro data. Across xenograft and syngeneic models of colorectal, hepatocellular, lung, breast, and bladder cancer, HDW-based interventions reduced tumor volume, tumor weight, or overall tumor burden relative to controls [12,19,42,44,48,51,65,66,67,72,73,82,84,91]. Polysaccharides, ethanol extracts, and aqueous ODE preparations produced marked tumor growth inhibition in mice, in some cases comparable to standard anticancer agents, and in leukemia models HDW increased survival while limiting splenomegaly [56,65,67,72]. Histological analyses showed reduced proliferation markers such as Ki-67 or PCNA and increased TUNEL-positive apoptotic cells in tumor tissue, confirming that macroscopic tumor control was accompanied by enhanced cell death [44,48,51,66,67,72,73,91]. Several studies simultaneously documented preserved body weight, stable liver and kidney function, and unremarkable organ histology, suggesting a favorable systemic safety profile at therapeutically active doses [12,19,44,51,65,67].

#### 3.3.4. Inhibition of Metastasis, Invasion, and Angiogenesis

HDW and its constituents also constrained metastatic behavior. Extracts and isolated compounds reduced migration and invasion in colorectal, hepatocellular, lung, and ovarian cancer models, frequently in parallel with the downregulation of epithelial-mesenchymal transition (EMT) and invasion related proteins such as N-cadherin, vimentin, MMP-2, MMP-9, and uPA, and the restoration of E-cadherin expression [26,50,51,59,66,72,73,74,83,89]. OD and HDW decreased lung metastatic nodules in animal models, while a suppression of wound closure and invasion was observed in zebrafish metastasis assays [59,86]. In a circulating tumor cell model, HDW reduced adhesion to the extracellular matrix, endothelial cells, and platelets, leading to a lower lung metastatic burden without affecting body weight [90]. Vascular modulation was another recurring feature: decreased tumor burden in some studies coincided with reductions in vascular markers such as CD31, α-SMA, and HIF-1α, indicating that anti-angiogenic effects and the remodeling of the tumor vasculature contribute to overall growth inhibition [19,84].

#### 3.3.5. Enhanced Sensitivity to Anticancer Therapies

A number of studies indicated that HDW-derived agents can act as chemosensitizers. Combinations of HDW or its constituents with cisplatin, 5-fluorouracil, arsenic trioxide, or HSP90 inhibitors produced a deeper suppression of proliferation, stronger apoptosis, and greater inhibition of colony formation than single-agent treatment [56,58,71,74,80,92]. HDW increased the responsiveness of colorectal and leukemia cells to cytotoxic drugs, and ursolic acid enhanced the antitumor efficacy of 5-fluorouracil with substantial reductions in tumor volume and weight in vivo [56,71]. Mechanistic work linked these synergistic effects to the modulation of signaling pathways associated with drug resistance and survival, including PI3K/AKT, ERK, STAT3, telomerase activity, β-catenin/CTNNB1, and DNA damage-response networks [22,55,62,67,71,80,82]. These findings suggest that HDW not only exerts direct antitumor actions but may also improve the therapeutic performance of existing anticancer regimens (Supplementary Materials Table S2).

### 3.4. Immune and TME Effects

#### 3.4.1. Immune Activation and Cytotoxic Enhancement

Evidence from preclinical studies indicates that HDW and its polysaccharide fractions augment antitumor immunity by strengthening effector lymphocyte function. In a syngeneic colorectal cancer model, polysaccharides from HDW markedly increased intratumoral CD4^+^ and CD8^+^ T-cell infiltration, accompanied by elevated levels of granzyme B, TNF-α, and IFN-γ, while reducing tumor burden [27]. The same study showed that these immune responses were further intensified when HDW polysaccharides were combined with PD-1 and CTLA-4 blockade, suggesting functional complementarity with immune checkpoint inhibition [27]. Immune cell-mediated cytotoxicity was also enhanced in adoptive cell therapy models. Treatment with HDW polysaccharides increased CD3^+^CD56^+^ effector populations and augmented TNF-α and IFN-γ production, resulting in superior tumor suppression in vivo when combined with cytokine-induced killer cells [70]. Alongside immune activation, improvements in systemic inflammatory status were observed in multiple animal models, with reductions in serum IL-6 and TNF-α reported in hepatocellular and colorectal cancer studies [12,44,46,48,84]. Collectively, these data indicate that HDW enhances antitumor immunity predominantly through the amplification of cytotoxic lymphocyte activity rather than the broad stimulation of suppressive immune subsets.

#### 3.4.2. Regulation of Inflammatory Signaling and Immunosuppressive Pathways

HDW consistently attenuated pro-tumorigenic inflammatory signaling across tumor models. Several studies documented the downregulation of key cytokines, including IL-6, IL-1β, IL-8, IL-17A, and TNF-α, indicating the inhibition of sustained inflammatory circuits that promote tumor progression [12,17,44,46,48]. These immunological changes were accompanied by the suppression of major regulatory pathways, such as NF-κB and JAK2/STAT3, which are central to tumor-associated immune evasion and cytokine production [48,55,84]. At the molecular level, ursolic acid blocked STAT3 phosphorylation and nuclear translocation and increased miR-4500 expression, thereby disrupting STAT3-driven oncogenic signaling [55]. Functional validation confirmed that miR-4500 inhibition partially reversed the antitumor effects of ursolic acid, directly linking STAT3 regulation to immune-modulatory and cytotoxic outcomes [55]. Quercetin similarly suppressed IL-6 and TNF expression at both transcript and protein levels while activating AGE-RAGE signaling, suggesting an additional immunoregulatory axis involved in HDW-mediated effects [17]. These findings support a dual role for HDW in dampening pathological inflammation while restoring immune competence within the TME.

#### 3.4.3. TME Remodeling and Metastatic Control

HDW and its constituents exerted pronounced effects on the structural and biochemical components of the TME. Multiple studies reported the reversal of epithelial-mesenchymal transition, reflected by an increased E-cadherin and decreased N-cadherin or vimentin expression, which translated into reduced migratory and invasive phenotypes in colorectal, hepatic, lung, and breast cancer models [43,57,59,64,69,86]. Coumarin polymers further potentiated these anti-EMT effects when co-administered with HDW, highlighting a cooperative mechanism in metastatic suppression [69]. Anti-angiogenic and anti-lymphangiogenic activity was also consistently observed. HDW downregulated VEGFA, VEGF-C, VEGFR3, CD31, and α-SMA expression and impaired endothelial tube formation, thereby inhibiting vascular and lymphatic support of tumor growth [63,84,87]. These vascular effects were accompanied by the suppression of PI3K/AKT, ERK, and STAT3 signaling cascades, indicating that angiogenic regulation is mechanistically linked to growth-factor pathway inhibition [63,84,87]. Metabolic remodeling constituted another dimension of microenvironmental control. HDW partially normalized tumor-induced metabolic disturbances in rats by correcting amino acid, glycolytic, and lipid profiles, suggesting the restoration of systemic metabolic balance in tumor-bearing hosts [79]. Ursolic acid further disrupted tumor metabolism by suppressing glycolysis through the SP1-dependent regulation of CAV1, with functional knockdown experiments confirming the CAV1/SP1 axis as critical for its metabolic and antiproliferative effects [81]. In metastatic models, HDW reduced circulating tumor cell adhesion to platelets and endothelial cells through the inhibition of Src/FAK signaling, leading to decreased pulmonary metastasis without causing body weight loss [90]. Ferroptosis-related microenvironmental changes were also reported, with increased lipid ROS, Fe^2+^ accumulation, and an upregulation of HMOX1 and transferrin receptor expression in lung cancer models [19]. Taken together, these results indicate that HDW reshapes the TME through the coordinated regulation of immune infiltration, inflammatory signaling, vascular support, metabolic pathways, and extracellular matrix dynamics, thereby creating conditions that are less permissive for tumor growth and dissemination (Supplementary Materials Table S3).

### 3.5. Network Pharmacology Analysis Results

#### 3.5.1. Related Targets of HDW in the Immune and TME Gene Sets

**Figure 2:**
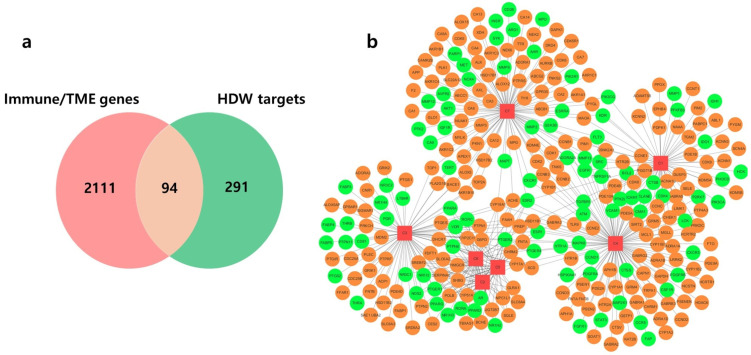
Immune- and tumor microenvironment-related targets of *Hedyotis diffusa* and the compound-target network. (**a**) Overlap between predicted targets of *Hedyotis diffusa* and immune- and tumor microenvironment-related gene sets. (**b**) Compound-target network of *Hedyotis diffusa*. Red squares represent active compounds, and circles represent predicted targets. Orange nodes indicate immune- and tumor microenvironment-related targets, and green nodes indicate non-related targets. Active compounds: C1, 2,3-dimethoxy-6-methyanthraquinone; C2, poriferasterol; C3, (4aS,6aR,6aS,6bR,8aR,10R,12aR,14bS)-10-hydroxy-2,2,6a,6b,9,9,12a-heptamethyl-1,3,4,5,6,6a,7,8,8a,10,11,12,13,14b-tetradecahydropicene-4a-carboxylic acid; C4, 2-methoxy-3-me-thyl-9,10-anthraquinone; C5, stigmasterol; C6, β-sitosterol; and C7, quercetin.

A total of 2110 immune- and TME-related targets were collected from the ImmPort (*n* = 1793) and GeneCards (*n* = 477) databases. From the TCMSP database, 37 compounds associated with HDW were initially retrieved, among which 7 compounds met the predefined pharmacokinetic criteria (OB ≥ 30% and DL ≥ 0.18). These active compounds corresponded to 291 predicted protein targets, of which 94 overlapped with the immune- and TME-related gene set (Figure 2a). The relationships between active compounds and their targets are illustrated as a compound-target network in Figure 2b. In the network, 417 red squares denote HDW-derived compounds and circles indicate predicted targets, with 418 green nodes representing immune- and TME-related genes and orange nodes indicating 419 non-immune/TME-related targets. Most targets in the network were associated with immune functions and microenvironmental regulation, supporting the involvement of 421 HDW in antitumor immunity and TME regulation.

#### 3.5.2. PPI Network Construction and Topological Analysis

**Figure 3:**
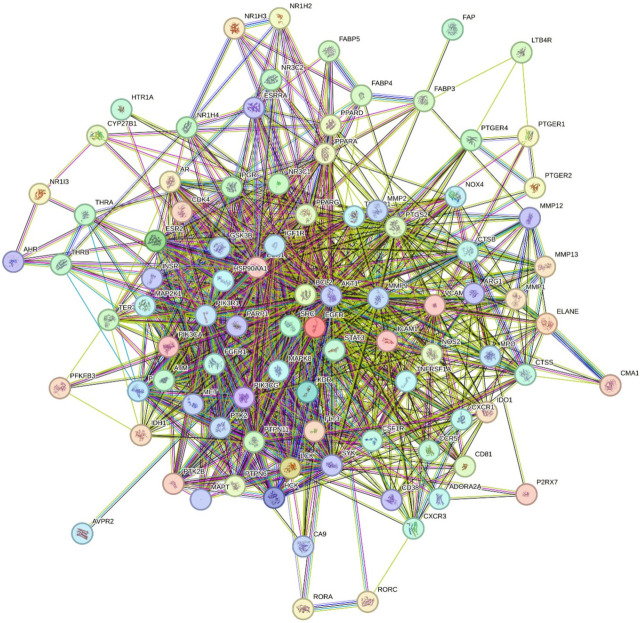
Computationally predicted protein-protein interaction network.

The intersecting immune- and TME-related targets of HDW were used to construct a PPI network based on STRING analysis (Figure 3). After the removal of isolated nodes, the final network consisted of 94 nodes and 1136 edges, with an average node degree of 24.2 and a clustering coefficient of 0.641, reflecting a highly interconnected network topology. Topological analysis identified several hub genes with a high degree centrality. AKT1, STAT3, EGFR, SRC, and BCL2 ranked as the most highly connected nodes, indicating their central positions within the network. Additional high-degree targets included MMP9, ESR1, PPARG, HSP90AA1, and PIK3R1, reflecting an involvement in pathways related to invasion, hormone signaling, inflammatory regulation, and cellular stress responses. Overall, the PPI structure demonstrated that HDW-related immune and TME targets form a dense interaction network characterized by multiple interconnected signaling hubs.

#### 3.5.3. Module Detection

**Figure 4:**
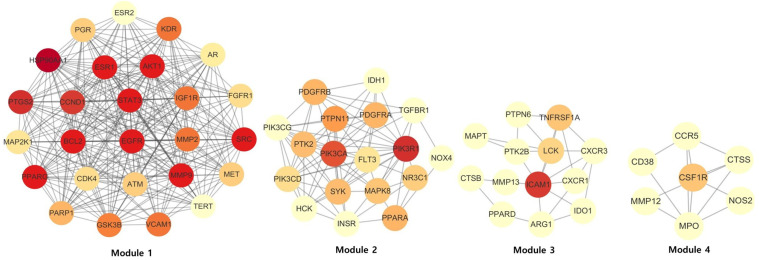
MCODE-based module clustering of the computationally constructed PPI network. Module 1: MCODE score = 22.56; Module 2: MCODE score = 9.647; Module 3: MCODE score = 4.167; and Module 4: MCODE score = 1.

Module analysis using the MCODE algorithm identified four highly interconnected clusters within the PPI network (Figure 4). Module 1 contained 26 nodes and was dominated by key regulators involved in cell survival, hormone signaling, and inflammatory control. Module 2 comprised 18 targets mainly associated with growth-factor signaling and PI3K-related pathways. Module 3 included 13 nodes enriched in immune signaling and cytokine-related processes. Module 4 consisted of seven targets primarily linked to innate immune responses and inflammatory remodeling. Collectively, these findings indicate that HDW-related targets are organized into functionally distinct sub-networks rather than forming a single homogeneous interaction network.

#### 3.5.4. Functional Enrichment Analysis of Intersecting Targets

**Figure 5:**
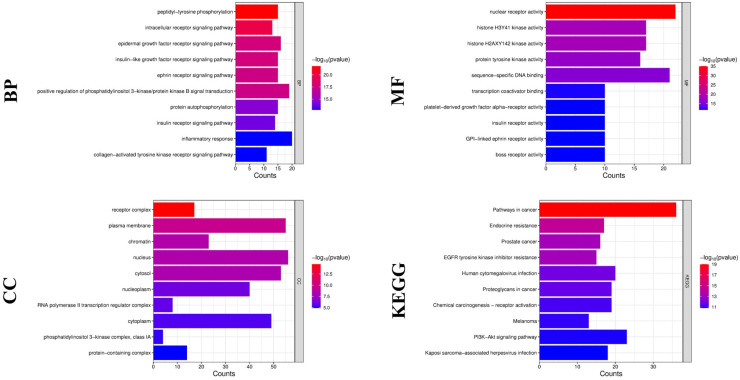
GO and KEGG enrichment analysis of the intersecting immune- and TME-related targets of HDW. Enrichment results are based on computational analysis of predicted targets rather than direct experimental validation. BP, biological process; CC, cellular component; MF, molecular function; GO, Gene Ontology; KEGG, Kyoto Encyclopedia of Genes and Genomes; TME, tumor microenvironment; HDW, *Hedyotis diffusa* Willd.

GO function enrichment and KEGG pathway enrichment analyses were performed for the 94 intersecting targets using the DAVID database (https://davidbioinformatics.nih.gov/ (accessed on 25 October 2025)). The GO analysis comprised biological processes (BP), cellular components (CC), and molecular functions (MF), and the results are summarized in Figure 5.

In the BP category, the intersecting targets were strongly enriched in peptidyl-tyrosine phosphorylation, intracellular receptor signaling, and multiple receptor tyrosine kinase-associated pathways, including epidermal growth factor receptor, insulin-like growth factor, ephrin, and insulin signaling. The positive regulation of PI3K-AKT signaling and inflammatory response also ranked among the most significant biological processes, indicating a convergence of growth signaling and immune-related mechanisms. In the CC category, the major enriched terms included plasma membrane, nucleus, cytosol, receptor complex, chromatin, and nucleoplasm, suggesting that the intersecting targets are spatially distributed across membrane signaling compartments and nuclear regulatory structures. In the MF category, nuclear receptor activity, protein tyrosine kinase activity, sequence-specific DNA binding, and transcription coactivator binding were predominant, highlighting roles in signal transduction and transcriptional regulation.

KEGG pathway analysis demonstrated a significant enrichment in pathways in cancer, endocrine resistance, prostate cancer, EGFR tyrosine kinase inhibitor resistance, and the PI3K-Akt signaling pathway. Additional enriched pathways involved proteoglycans in cancer, focal adhesion, AGE-RAGE signaling, central carbon metabolism in cancer, and prolactin signaling, reflecting the integrated regulation of oncogenic signaling, metabolism, and microenvironmental interaction. Together, these findings indicate that HDW-related targets are concentrated within receptor tyrosine kinase-centered signaling axes and PI3K-AKT-dominated networks, linking immune regulation, metabolic control, and tumor progression through a coordinated multi-pathway framework.

## 4. Discussion

**Figure 6:**
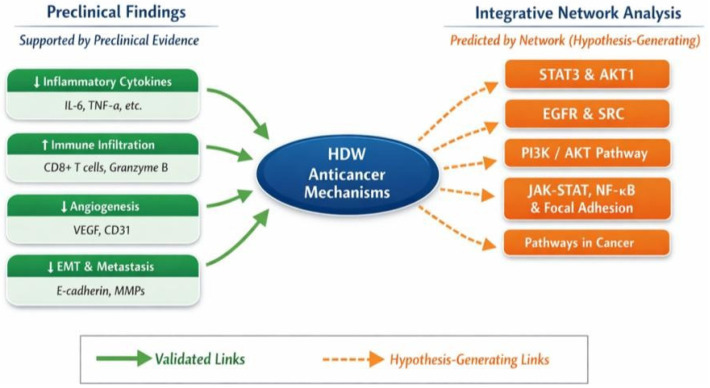
Integration of preclinical evidence with network pharmacology predictions.

The present study brings together the broad preclinical literature and a systems-level network analysis to clarify how HDW modulates antitumor immunity and the TME. Contemporary cancer biology views tumors as dynamic ecosystems in which malignant cells, stromal elements, immune populations, and vascular structures co-evolve through chronic inflammation and immune escape [93]. Within this framework, the TME is not only a barrier to therapy but also a source of therapeutic vulnerabilities. Natural products remain a major reservoir of multi-target scaffolds for oncology drug discovery, and several have already entered clinical use [9,10]. Previous reviews have summarized the chemistry and broad pharmacology of HDW but have not focused systematically on immune and TME mechanisms [14,15]. By structuring heterogeneous preclinical findings and embedding them within a network of immune- and TME-related targets, our work reframes HDW as a candidate multi-component modulator of tumor ecology rather than a single-pathway cytotoxic agent. Network-based analyses are used to contextualize, rather than replace, the experimentally derived findings (Figure 6).

Across tumor types, the scoping review shows that growth inhibition and regulated cell death are among the most consistent effects of HDW. Extracts, polysaccharides, flavonoid-rich fractions, and defined constituents such as quercetin and ursolic acid reduce proliferation, induce cell cycle arrest, and trigger apoptosis or ferroptosis in colorectal, cervical, hepatocellular, lung, and prostate cancer models [11,12,16,19,42,44,48,51]. These phenotypes are accompanied by the modulation of cyclins, CDKs, Bcl-2 family proteins, and ferroptosis-related markers, suggesting that HDW interferes with survival and stress-response programs at multiple points. The network analysis helps to rationalize this diversity: intersecting immune/TME targets cluster around hub genes such as AKT1, STAT3, BCL2, and EGFR within a dense PPI network. The enrichment of PI3K-Akt-centered “Pathways in cancer” indicates that relatively few highly connected nodes may integrate upstream compound signals into broad effects on proliferation and cell death.

In vivo experiments support the translation of these molecular changes into macroscopic tumor control with an acceptable systemic safety. HDW-based preparations consistently reduced tumor volume or weight in xenograft and syngeneic models of colorectal, liver, lung, and bladder cancer, with corresponding decreases in proliferation markers and increases in apoptotic indices in tumor tissue [19,42,51,84]. Multiple studies reported preserved body weight and a largely unremarkable histology of major organs, suggesting a therapeutic window in preclinical systems [42,51,67]. At the same time, HDW enhanced the efficacy of conventional agents such as cisplatin and arsenic trioxide, producing a deeper suppression of proliferation and apoptosis induction than monotherapy [56,74]. In the network, these observations align with modules enriched for growth-factor and PI3K-related signaling, in which AKT1, EGFR, and related kinases act as central nodes that regulate both intrinsic survival pathways and responses to cytotoxic or targeted therapy [31,37,40]. 

The immune findings reveal that HDW not only affects tumor cells directly but also alters antitumor immunity. Polysaccharide-rich preparations increased intratumoral CD4^+^ and CD8^+^ T-cell infiltration, enhanced granzyme B, TNF-α, and IFN-γ production, and improved the performance of immune checkpoint blockade and adoptive cell therapy in colorectal cancer models [17,27,70]. Parallel reductions in IL-6, TNF-α, and other pro-inflammatory cytokines, together with the inhibition of NF-κB and JAK2/STAT3 signaling, indicate the suppression of chronic tumor-promoting inflammation [17,25,48]. Our immune- and TME-focused gene sets from ImmPort and GeneCards captured this duality by highlighting targets involved in cytokine signaling, innate immunity, and inflammatory remodeling. STAT3 and related transcriptional regulators emerged as hubs that connect inflammatory pathways with survival signaling, which may provide a plausible mechanistic context for improved cytotoxic responses and dampened pathological inflammation in the TME [17,31].

The remodeling of the structural and metabolic components of the TME is another recurring theme that becomes clearer when mapped onto network modules. Experimentally, HDW and its constituents reversed epithelial-mesenchymal transition, reduced migration and invasion, and downregulated EMT-related proteins while restoring E-cadherin in colorectal and lung cancer models [43,69,86]. Anti-angiogenic and anti-lymphangiogenic effects were demonstrated through reduced VEGF-related markers and impaired endothelial tube formation in liver and colorectal cancer [19,63,84]. Metabolomics work further showed a partial normalization of tumor-induced disturbances in amino acid, glycolytic, and lipid pathways in tumor-bearing rats [79]. Within the network, the enrichment for focal adhesion, proteoglycans in cancer, and central carbon metabolism suggests that HDW influences how tumor cells adhere, signal through the matrix, and utilize nutrients. Together, these data support a model in which HDW reshapes the microenvironmental “soil” that supports tumor “seeds,” making it less permissive for invasion, vascularization, and systemic metabolic disruption.

The compound-target network underscores that these effects arise from cooperation among multiple HDW constituents rather than the action of a single molecule. A subset of compounds that passed pharmacokinetic filters yielded a compact yet densely connected network of immune- and TME-related targets, in which flavonoids and triterpenoids such as quercetin and ursolic acid occupy central positions. Independent studies have shown these molecules to modulate STAT3, PI3K-Akt, and metabolic pathways in colorectal and other cancers, supporting their roles as key drivers within the HDW pharmacological profile [45,55,88]. Network pharmacology analyses in colorectal and prostate cancer similarly highlight PI3K-Akt, MAPK, and matrix-related pathways as recurrent nodes of HDW action [23,24]. By integrating our scoping review with these in silico results, HDW can be viewed as a modular assembly of compounds acting on overlapping sub-networks controlling cell survival, immunity, and tissue architecture.

From a translational viewpoint, this integrated picture positions HDW as a candidate adjunct for strategies that target the TME and antitumor immunity. The ability of HDW preparations to enhance checkpoint blockade, augment adoptive cell therapies, and sensitize tumors to chemotherapy resonates with current efforts to exploit microenvironmental vulnerabilities and overcome immune evasion [4,5,17]. At the same time, the evidence base is almost entirely preclinical and is characterized by a substantial heterogeneity in models, formulations, and dosing. In addition, common internal validity limitations, such as the frequent non-reporting of sample size, incomplete reporting of randomization or blinding, and variable characterization of HDW preparations across different plant species, plant parts, and developmental stages, may introduce bias and limit comparability across studies. These limitations reflect an inherent preclinical bias, and the lack of a consistent pharmacokinetic characterization of HDW preparations and their key constituents, including bioavailability, metabolism, and tissue distribution, further constrains translational inference.

As a scoping review, our work maps this landscape but does not provide pooled effect estimates or formal risk-of-bias assessments. On the network side, target prediction and enrichment depend on available databases and may miss relevant interactions. In addition, database-driven target prediction introduces uncertainty and does not account for exposure-related constraints such as bioavailability, metabolism, or tissue distribution. Therefore, the in silico results should be interpreted as hypothesis-generating rather than causal. Because the network component was intentionally centered on shared immune- and TME-related hubs across heterogeneous preclinical tumor models, we did not perform a compound-disease/drug-disease pathway analysis for specific cancer indications, and disease-specific interpretations should therefore be viewed accordingly. Future work should therefore combine standardized in vivo experiments with a focused validation of priority hubs such as AKT1, STAT3, and caveolin-1, extend the network framework to cancer-type-stratified compound-target-disease/pathway analyses, evaluate rational combinations with immunotherapy, and eventually link HDW exposure to defined immune and TME endpoints with biomarker-guided stratification in early-phase clinical settings.

## 5. Conclusions

This study integrates a scoping review of preclinical evidence with network pharmacology to clarify how HDW influences antitumor immunity and the TME. Across diverse cancer models, HDW consistently suppresses tumor growth while modulating immune activity, inflammatory signaling, angiogenesis, epithelial-mesenchymal transition, and metabolic pathways. A systems-level analysis indicates that these heterogeneous effects converge on a limited set of highly connected signaling hubs, particularly the PI3K-Akt-STAT3 axis, linking direct cytotoxic stress with immune activation and microenvironmental remodeling. Rather than acting as a single-pathway agent, HDW emerges as a multi-component botanical modulator that reshapes tumor-immune interactions and the surrounding microenvironment. Although the current evidence is largely preclinical and exploratory, this integrated framework provides a rationale for the further investigation of HDW as a potential adjunct strategy in preclinical and translational research targeting immune and microenvironmental vulnerabilities in cancer.

## Figures and Tables

**Table 1:** Characteristics of the included preclinical studies.

Study ID	Study Design	Cancer Type	Experimental Model	Sample Size	Measured Outcomes
Bai 2025a (China) [42]	In vitro + In vivo	Bladder cancer	Human BLCA cells + nude mice xenograft	In vitro: NR/ In vivo: *n* = 6 per group	1. qPCR: GPX4, SLC7A11, FTH1, COX2, TFRC, HMOX1 mRNA; 2. Western blot: GPX4, FTH1, TFRC; 3. Tumor volume & tumor weight; 4. Ki67 immuno-fluorescence; 5. mRNA/protein levels of CAV1, JUN, VEGFA
Bai 2025b (China) [27]	In vivo	Colorectal cancer	C57BL/6 mice (MC38 model) and BALB/c mice (CT26 model)	MC38: 4 groups (*n* = 8 each); CT26: 4 groups (*n* = 8 each)	1. Tumor volume and tumor weight; 2. CD4+ and CD8+ T-cell infiltration (IHC); 3. Cytotoxic T-cell effectors (GZMB, TNFα, IFN-γ); 4. Flow cytometry: CD8+, GZMB+, TNFα+, IFN-γ+ cells; 5. Immune profiling (Treg, B cells, NK cells, DCs, macrophages)
Chen 2018 (China) [43]	In vitro	Colorectal cancer	Human colorectal cancer HCT-8 cells	NR	1. Cell viability (MTT); 2. Cell density (microscopy); 3. Migration (Transwell); 4. Invasion (Transwell-Matrigel); 5. EMT proteins (E-cadherin, N-cadherin, Vmentin); 6. TGF-β/Smad proteins (TGF-β, p-Smad2/3, Smad4)
Chen 2022 (China) [44]	In vitro + In vivo	Hepatocellular carcinoma	HCC cell lines (HepG2, Hep3B, HCCLM3, H22) + BALB/c nude mice xenograft	In vitro: NR; In vivo: Control *n* = 6, Model *n* = 4, FOD *n* = 5	1. Cell viability (CCK-8); 2. Ki67 expression; 3. Cell cycle (PI-FACS); 4. Apoptosis (Annexin V/PI); 5. Cleaved caspase-3 (FACS/WB); 6. Autophagy markers LC3B-II, P62; 7. Autophagic flux (MDC, LysoTracker, mCherry-GFP-LC3B); 8. ER stress markers p-PERK, p-EIF2α, ATF4, CHOP; 9. ROS levels; 10. Tumor volume/weight; 11. Serum IL-6, TNF-α; 12. ALT, AST, UREA, CREA
Chen 2025 (China) [45]	in vitro	Breast cancer	Human breast cancer cell lines (MCF-7, MDA-MB-231)	NR	1. Cell viability (MTT); 2. BIRC5 mRNA; 3. CDK1 mRNA; 4. FOS mRNA; 5. HSP90AA1 mRNA; 6. BIRC5 protein; 7. CDK1 protein; 8. HSP90α protein
Cheng 2022 (China) [46]	In vitro + In vivo	Hepatocellular carcinoma	Male C57BL/6 mice on high-fat diet (HFD) + hepatocarcinogenesis model; AML12 hepatocytes	In vitro: *n* = 3; In vivo: *n* = 8/group	1. Serum ALT/AST; 2. Liver triglycerides (TG); 3. Histology (HE, Oil Red O); 4. Tumor number & size; 5. Inflammatory cytokines (TNF-α, IL-6); 6. Oxidative stress (ROS, MDA); 7. SIRT1, p53, NF-κB p65 protein expression; 8. Ki67 and α-SMA (IHC)
Cheng 2025 (China) [13]	In vitro	Hepatocellular carcinoma	Human HCC cells (Huh- 7) + normal liver cells (WRL68)	In vitro: *n* = 3 per experiment (reported); no animal model used	1. Cellular uptake (PKH26); 2. Cell viability (CCK-8); 3. Proliferation (cell counting); 4. Morphological changes; 5. Apoptosis (Annexin V/PI); 6. Cell cycle distribution; 7. RNA-seq transcriptomics; 8. qRT-PCR (PI3K, AKT, mTOR, Bcl- 2, p53, Bax, Caspase-3/8/9); 9. Western blot (p53, Caspase-3/8/9)
Chung 2017 (Republic of Korea) [47]	In vitro + In vivo	Colorectal cancer	Human CRC HT-29 cells + BALB/c nude mice xenograft	In vitro: *n* = 3; In vivo: *n* = 6/group	1. Cell viability (MTT); 2. Apoptosis (Annexin V/PI); 3. ROS generation (DCFDA); 4. Mitochondrial membrane potential (JC-1); 5. Western blot (Bax, Bcl-2, caspase-3, caspase-9, cytochrome c); 6. Tumor volume & weight; 7. Histology (H&E)
Feng 2017 (China) [48]	In vitro + In vivo	Colorectal cancer	Human CRC cell lines; BALB/c nude mice (HT-29 xenograft)	In vitro: NR; In vivo: *n* = 10/group	1. Cell viability (MTS); 2. Tumor volume/weight; 3. Ki-67; 4. TUNEL; 5. Western blot (cytochrome c, caspase-3, caspase-9, PARP); 6. IHC (Pim-1, Bcl-2, Bax, COX-2, iNOS, eNOS, HIF-1α); 7. Serum cytokines (IL-1β, IL-6, TNF-α, IL-4, IL-10); 8. Phosphoproteins (p-AKT, p-Erk1/2, p-JNK, p-p38, p-p70S6K, p-STAT3, p-p53)
Feng 2025 (China) [11]	In vitro + In vivo	Prostate cancer	Human prostate cancer cell lines (RM1, LNCaP); BALB/c nude mice xenograft	In vitro: NR; In vivo: *n* = 6/group	1. Cell viability (MTT, CCK-8); 2. Colony formation; 3. Apoptosis (flow cytom- etry); 4. TUNEL; 5. Migration/invasion (Transwell); 6. Western blot (PIAS4, STAT3, p-STAT3, DDB2, AR); 7. STAT3 activity assay; 8. ChIP (STAT3-DDB2 promoter binding); 9. Co-IP (AR ubiquitination); 10. IHC (PIAS4, AR, Ki67)
Han 2023 (China) [49]	In vitro	Malignant glioma	Human malignant glioma cell lines; U87 3D spheroids in microfluidic chip	NR	1. Cell viability (MTT); 2. Apoptosis (Annexin V/PI); 3. Scratch migration assay; 4. Transwell migration; 5. Transwell invasion; 6. 3D microfluidic invasion (vimentin fluorescence); 7. Network pharmacology (MAPK, Wnt, cytoskeleton pathways)
Ho 2018 (Malaysia) [50]	In vitro	Colorectal cancer	Human CRC cell lines (HCC2998, KM12)	NR	1. Cell viability (MTT); 2. Migration (wound healing); 3. Colony number; 4. Colony area
Huang 2021 (China) [51]	In vitro + In vivo	Hepatocellular carcinoma	Human SMMC-7721, SK- hep1, HepG2 cell lines + nude mouse xenograft	In vitro: NR; In vivo: *n* = 12 (6/group)	1. Cell viability (MTT, CCK-8); 2. Colony formation; 3. Migration (Transwell); 4. WB: p-AKT, p-mTOR, p-ERK, p-4EBP1; 5. WB: Bcl-2, Bax; 6. Tumor volume & tumor weight; 7. Body weight; 8. IHC: Ki67; 9. Organ histology (H&E liver/kidney)
Huang 2022 (China) [19]	In vitro + In vivo	Lung cancer	A549 and H1975 human lung adenocarcinoma cell lines + BALB/c nude mouse xenograft	In vitro: NR; In vivo: *n* = 15	1. Cell viability (CCK-8); 2. Colony formation; 3. Migration & invasion (Transwell, wound healing); 4. TEM mitochondrial morphology; 5. Lipid ROS (BODIPY-C11); 6. Fe^2+^ staining; 7. MDA level; 8. Western blot: GPX4, VDAC2/3, Bax, Bcl-2, HMOX1, TFR; 9. Ferroptosis blocking (OE-GPX4, zileuton, si-VDAC2/3); 10. Tumor volume & weight; 11. Organ histology (H&E); 12. IHC: Bax, Bcl-2, VDAC2/3, 4-HNE, TFR, HMOX1
Jiang 2017 (China) [52]	In vitro	Myelodysplastic syndrome	SKM-1 human MDS leukemia cells	NR	1. Cell viability (MTT); 2. Morphological apoptosis (Hoechst 33258); 3. Apoptosis (Annexin V/PI); 4. Western blot: caspase-3/8/9, PARP; 5. Western blot: PI3K, Akt, p-Akt, p-P65
Jin 2022 (China) [53]	In vitro	Gallbladder carcinoma	GBC-SD murine gallbladder carcinoma cell line	NR	1. Apoptosis (flow cytometry); 2. Migration (wound healing); 3. Invasion (Transwell); 4. Differentially expressed proteins (proteomics); 5. Pathway regulation (PI3K-Akt; Wnt; HIF-1; focal adhesion; microRNAs in cancer); 6. TME- related cytokines (IL-8) and ECM/motility proteins
Jing 2023 (China) [54]	In vitro + In vivo	Osteosarcoma	Human MNNG/HOS and U-2 OS cell lines + nude mouse xenograft	NR	1. Cell viability (CCK-8); 2. Colony formation; 3. Apoptosis (Annexin V/PI, WB); 4. Migration and invasion (Transwell); 5. Tumor volume and tumor weight (xenograft); 6. Ki-67 IHC; 7. RNA-seq DEGs; 8. MYC mRNA & protein levels; 9. CHK1 and RAD51/p-RAD51 levels; 10. γ-H2A.X nuclear localization; 11. IC50 of DDR inhibitors (olaparib, NU7441, RI-1, AZD7762, MK2206); 12. Rescue assays (MYC overexpression/si-MYC, SC79, MK2206)
Kim 2018 (Republic of Korea) [55]	In vitro	Colorectal cancer	Human HCT116 and HT29 cell lines	NR	1. Cell viability (MTT); 2. Apoptosis (TUNEL); 3. Sub-G1 population (flow cytometry); 4. PARP and caspase-3 cleavage (WB); 5. JAK2/STAT3 phosphorylation (WB); 6. STAT3 nuclear translocation (IF); 7. miR-4500 expression (qRT-PCR); 8. Effects of miR-4500 inhibitor on cytotoxicity, colony formation, apoptosis, and p-STAT3
Kuo 2017 (Taiwan) [56]	In vitro + In vivo	Acute promyelocytic leukemia	WEHI-3 murine leukemia cells + BALB/c leukemia mouse model	In vitro: NR; In vivo: 9 groups × *n* = 6 each	1. Cell viability (Prestoblue assay); 2. Apoptosis (caspase cascade, PARP cleavage); 3. DR4/DR5 protein expression; 4. Bcl-2 family protein levels (Bcl-2, Bcl-xL, survivin, Bak, Bid/t-Bid); 5. Spleen and liver weight (leukemic mice); 6. Survival rate of tumor-bearing mice
Lai 2017 (China) [57]	In vitro	Colorectal cancer	HCT-8/5-FU multidrug-resistant CRC cell line	NR	1. Cell viability (MTT); 2. Migration (wound healing); 3. Migration & invasion (Transwell); 4. Adhesion assay; 5. mRNA levels of TGF-β, SMAD4, E-cadherin, N-cadherin (RT-sqPCR); 6. Protein expression of TGF-β, SMAD4, E-cadherin, N-cadherin (Western blot)
Lee 2016 (Republic of Korea) [58]	In vitro	Colorectal cancer	Human HT-29 cell line	NR	1. Cell viability; 2. Sub-G1 apoptotic population; 3. Mitochondrial membrane depolarization; 4. Caspase-3/-9 activity; 5. Intracellular ROS generation; 6. Chemosensitivity to paclitaxel/5-FU/cisplatin/etoposide/doxorubicin/docetaxel
Lee 2019 (Republic of Korea) [59]	In vitro + In vivo	Colorectal cancer	HCT116 and SW480 human CRC cell lines + BALB/c nude mice xenograft	NR	1. Cell viability; 2. Migration (wound healing); 3. Invasion (Transwell); 4. EMT marker expression (E-cadherin, N-cadherin, vimentin); 5. AMPK phosphorylation; 6. Lung metastasis in vivo
Li 2016 (China) [60]	In vitro	Hepatocellular carcinoma	Human HepG2 cell line	NR	1. Cell viability (SRB); 2. Apoptosis (AO/EB, Annexin V/PI, DNA ladder); 3. Caspase-3/8/9 activities; 4. Mitochondrial membrane potential; 5. ROS levels; 6. Protein expression (p53, Bax, Bcl-2, cytochrome C, Fas, FasL, p21, cyclin E, CDK2); 7. mRNA expression (p53, Bax, Bcl-2); 8. Cell cycle distribution
Li 2017 (China) [61]	In vitro	Lung cancer	NCI-H460 human lung cancer cell line	NR	1. Apoptotic morphology (Hoechst33342/PI staining); 2. Apoptosis rate (flow cytometry); 3. Survivin protein expression (Western blot); 4. Livin protein expression (Western blot)
Li 2018 (China) [62]	In vitro	Colorectal cancer	HCT-8/5-FU human CRC cells	NR	1. Cell viability (MTT); 2. Colony formation; 3. Apoptosis (Annexin V/PI, DAPI morphology); 4. mRNA of Bcl-2, Bax, Cyclin D1, CDK4, p21; 5. Protein of Bcl-2, Bax, Cyclin D1, CDK4, p21; 6. PI3K/AKT pathway proteins (PTEN, PI3K, AKT, p-AKT)
Li 2019 (China) [63]	In vitro	Colorectal cancer	HCT116 andHCT-8 CRC cells + VEGF-C-stimulated HLECs	NR	1. CRC cell viability (MTT); 2. CRC colony formation; 3. CRC migration (wound healing, Transwell); 4. VEGF-C expression & secretion (WB, ELISA); 5. HLEC viability; 6. HLEC colony formation; 7. HLEC cell cycle; 8. HLEC apoptosis; 9. HLEC migration (Transwell); 10. HLEC tube formation; 11. MMP2, MMP9, cyclin D1, CDK4 (WB); 12. Signaling: VEGFR3, PI3K/p-PI3K, AKT/p-AKT, ERK/p-ERK, STAT3/p-STAT3
Lin 2019a (China) [64]	In vitro	Lung cancer	Human A549 NSCLC cell line	NR	1. Adhesion assay; 2. Migration (Transwell); 3. Invasion (Transwell + Matrigel); 4. MMP-2 & MMP-9 activities (zymography); 5. MMP-2 & MMP-9 protein expression; 6. TIMP-1 & TIMP-2 activities (reverse zymography); 7. TIMP-1 & TIMP-2 protein expression; 8. EMT markers (E-cadherin, N-cadherin, vimentin); 9. COX-2 expression; 10. EGFR/p-EGFR; 11. PI3K/Akt signaling (Akt, p-Akt); 12. MAPK signaling (ERK1/2, p-ERK1/2, JNK, p-JNK, p38, p-p38)
Lin 2019b (China) [65]	In vitro + In vivo	Lung cancer	Human A549 cell line + BALB/c nude mouse xenograft	In vitro: NR; In vivo: *n* = 40 (10/group)	1. Cell viability (MTT) in A549 and WI38; 2. Colony formation; 3. Apoptosis (Annexin V/PI); 4. Caspase-9 and caspase-3 activity; 5. Cytochrome c cytosolic release; 6. Bax and Bcl-2 protein expression; 7. Bax/Bcl-2 ratio; 8. Tumor volume (days 0, 5, 10, 15); 9. Tumor weight and inhibitory rate in xenograft model
Ling 2023 (China) [66]	In vitro + In vivo	Gastric cancer	MKN-45 and AGS human gastric cell lines + BALB/c nude mouse xenograft	In vitro: *n* = 3 per assay; In vivo: *n* = 6 per group	1. Cell viability (MTT); 2. Apoptosis (Hoechst 33342, Annexin V/PI); 3. Cell cycle (G1/S) distribution; 4. ROS production (DCFH-DA); 5. Mitochondrial membrane potential (JC-1); 6. Protein levels of Bax, Bcl-2, Apaf-1, Pro-caspase-9, Cleaved-caspase-3, Cytochrome C; 7. Tumor volume/weight in xenograft; 8. PCNA and Ki-67 expression (IHC); 9. Serum CA72-4
Lu 2016 (China) [67]	In vitro + In vivo	Colorectal cancer	HCT-116, DLD-1, HT-29, Lovo cell lines + primary CRC cells + HCT-116 SCID xenograft	In vitro *n* = 5 per assay; In vivo *n* = 10 per group	1. Cell viability (MTT); 2. Colony formation; 3. BrdU incorporation; 4. Cell death (Trypan blue); 5. Apoptosis (caspase-3 activity, histone-DNA ELISA, Annexin V/PI, TUNEL); 6. Cleaved PARP and cleaved caspase-3 (WB); 7. AMPK pathway (p-AMPK, p-ACC); 8. mTORC1 pathway (p-S6K1, Bcl-2, HIF-1α); 9. AMPK-p53 complex (Co-IP) and p53 activation (p-Ser15); 10. Genetic inhibition of AMPK or p53 (shRNA, dn-AMPK) effects on ODE response; 11. Tumor volume, tumor weight, tumor daily growth; 12. Tumor p-AMPK, p-ACC, p-p53, p-S6K1 in xenografts
Lv 2021 (China) [68]	In vitro	Multiple cancers	Human MCF-7, HepG2, A549, A2780 cell lines	NR	1. Cytotoxicity (MTT assay) and IC50 values in MCF-7, HepG2, A549, A2780
Lv 2023 (China) [69]	In vitro	Lung cancer	A549 human lung cancer cell line	NR	1. Migration (Transwell); 2. Invasion (Transwell); 3. EMT-related gene expression (E-cadherin, N-cadherin, vimentin) by RT-PCR
Ma 2019 (China) [70]	In vitro + In vivo	Hepatocellular carcinoma	Human PBMC-derived CIK cells + tumors (A549, MCF-7, HCT116 cell lines) + nude mouse xenograft	In vitro: *n* = 3; In vivo: *n* = 5/group	1. CD3 + CD56+ CIK proportion; 2. TNF-α+ and IFN-γ+ CIK cells; 3. CIK apoptosis; 4. Cytotoxicity vs. Hep3B/A549/MCF-7/HCT116; 5. CR3 expression & blocking assay; 6. Tumor volume/weight in vivo
Ma 2022 (Taiwan) [71]	In vitro + In vivo	Gastric cancer	AGS, SCM1, MKN45 human gastric cell lines + BALB/c xenograft	In vitro: NR; In vivo: TG: UA *n* = 7/CG: placebo *n* = 6/5- FU *n* = 5	1. Cell viability; 2. Colony formation; 3. CYP19A1 (aromatase) mRNA; 4. Ar protein; 5. UA + 5-FU synergy (CI); 6. Tumor volume (in vivo); 7. Tumor weight (in vivo)
Ning 2022 (China) [72]	In vitro + In vivo	Pancreatic cancer	PANC-1 and SW1990 human pancreatic cancer cell lines + BALB/c nude mouse xenograft	In vitro: NR; In vivo: *n* = 6/group	1. Cell viability (CCK-8); 2. Colony formation; 3. Apoptosis (Annexin V/PI); 4. Cell cycle (flow cytometry); 5. Migration (wound healing); 6. Invasion (Transwell); 7. ROS levels; 8. Mitochondrial membrane potential (JC-1); 9. Western blot: Bcl-2, Bax, cleaved caspase-3, PARP, p-Akt, Akt, p-ERK, ERK; 10. Tumor volume & weight (in vivo); 11. Ki-67 IHC; 12. TUNEL apoptosis in tumors
Ou 2024 (China) [73]	In vitro + In vivo	Lung cancer	A549 and H1975 human NSCLC cells + BALB/c nude mouse xenograft	In vitro: NR; In vivo: *n* = 6/group	1. Cell viability (CCK-8); 2. Colony formation; 3. Apoptosis (Annexin V/PI); 4. ROS levels; 5. Mitochondrial membrane potential (JC-1); 6. Migration (wound healing); 7. Invasion (Transwell); 8. Western blot: Bax, Bcl-2, cleaved caspase- 3, PARP, p-Akt/Akt, p-ERK/ERK; 9. Tumor volume & weight; 10. Ki-67 IHC; 11. TUNEL apoptosis in tumor tissue
Pu 2016 (China) [74]	In vitro	Osteosarcoma	MG-63 human osteosarcoma cells	NR	1. Cell viability (MTT); 2. Cell cycle distribution (flow cytometry); 3. Apoptosis (Annexin V/PI); 4. Migration (scratch assay); 5. Invasion (Transwell); 6. Western blot: Bax, Bad, Bcl-xl, Bcl-2, caspase-3, caspase-8, PARP
Sun 2016 (China) [75]	In vitro	Colorectal cancer	HT-29 CSC side population cells	NR	1. SP proportion (FACS); 2. Lgr5 protein expression (WB); 3. Sphere formation; 4. Cell viability (WST-1); 5. Morphological changes (phase contrast); 6. mRNA levels of ABCB1, β-catenin, c-Myc, PCNA, survivin (RT-PCR)
Trang 2025 (Vi- etnam) [76]	In vitro	Multiple cancers	MCF-7, SK-LU-1, HepG2 human cancer cell lines	NR	1. Cell viability (IC50 values); 2. DPPH antioxidant activity; 3. NO inhibition (RAW264.7); 4. α-glucosidase inhibition; 5. Antimicrobial MIC (*B. subtilis*, *S. aureus*, *E. coli*, fungi)
Wang 2017 (China) [77]	In vitro	Multiple cancers	HL-60, HeLa, HCT15, A549, HepG2, PC-3, CNE- 2, BGC-823 cell lines	NR	1. IC50 values for 10 isolated compounds across 8 tumor cell lines
Wang 2018a (China) [78]	In vitro	Multiple cancers	HeLa, HL-60, A549, HepG2, BGC-823, CNE-2, HCT15, PC-3 cell lines	NR	1. IC50 values against HeLa; 2. IC50 against HL-60; 3. IC50 against A549; 4. IC50 against HepG2; 5. IC50 against BGC-823; 6. IC50 against CNE-2; 7. IC50 against HCT15; 8. IC50 against PC-3
Wang 2018b (China) [79]	In vivo	Walker carcino- sarcoma	Walker-256 tumor in Wistar rats	*n* = 6 per group	1. Urine metabolomic biomarkers (NMR); 2. Plasma metabolomic biomarkers (NMR); 3. Tumor weight and metabolic pathway disturbances
Wang 2021a (China) [80]	In vitro	Myelodysplastic syndrome	SKM-1 human MDS cell line	NR	1. Cell proliferation (CCK-8 IC50); 2. Telomerase activity (TRAP-ELISA); 3. HSP90 mRNA expression (RT-qPCR); 4. Apoptosis rate (Annexin V/PI flow cytometry); 5. Apoptosis-related proteins (hTERT, cleaved caspase-3, cleaved caspase-8, cleaved PARP)
Wang 2021b (China) [81]	In vitro + In vivo	Breast cancer	MCF-7, MDA-MB-231, 4T1, 4T1-Luc cell lines + BALB/c mouse xenograft	In vitro: NR; In vivo: NR	1. Cell viability; 2. Breast cancer cell migration/invasion; 3. Glycolysis markers (glucose uptake, lactate production); 4. Protein expression (Cav-1, SP1); 5. siRNA knockdown (CAV1, SP1); 6. Tumor growth and metastasis in vivo
Wang 2023 (China) [82]	In vitro + In vivo	Lung cancer	A549 and H1299 NSCLC cell lines + A549-Luc nude mouse xenograft	In vitro: NR; In vivo: 3 groups (Control, 20 mg/kg, 60 mg/kg), *n* = NR	1. Cell viability (CCK-8, colony formation); 2. EdU proliferation; 3. Apoptosis (Annexin V-FITC flow cytometry); 4. Autophagic flux (mCherry-EGFP-LC3); 5. TEM autophagosome count; 6. WB: LC3B, Beclin-1, p62; 7. WB/qPCR: MET, p-MET; 8. WB: PI3K/p-PI3K, AKT/p-AKT, mTOR/p-mTOR; 9. Autophagy inhibition assays (3-MA, autophinib, si-Beclin1); 10. Xenograft tumor weight Volume, bioluminescence; 11. Tumor IHC (LC3B, Beclin-1, MET, p-PI3K, p-AKT, p-mTOR)
Wang 2024 (China) [22]	In vitro	Lung cancer	A549 human LUAD cell line	NR	1. Cell viability (CCK-8); 2. Apoptosis (Annexin V-FITC/PI); 3. CTNNB1 protein expression (Western blot); 4. CTNNB1 mRNA expression (qPCR)
Wu 2017 (China) [83]	In vitro	Laryngeal squamous cell carcinoma	Hep2 human LSCC cell line	NR	1. Cell viability (MTT); 2. Cell cycle distribution (PI-FACS); 3. Apoptosis (Annexin V-FITC/PI); 4. Caspase-3/8/9 protein levels (WB); 5. Bcl-2 protein (WB); 6. Cell invasion (Transwell); 7. MMP-2 and uPA protein expression (WB)
Wu 2022 (China) [26]	In vitro	Lung cancer	H1975 human NSCLC cells	NR	1. Cell viability (MTT); 2. Cell cycle distribution (PI-FACS); 3. Ki67 immuno-fluorescence; 4. Apoptosis (Annexin V/PI); 5. Bax, Bcl-2, Caspase-3/cleaved Caspase-3 (WB); 6. Migration (scratch); 7. MMP-2, MMP-9, TIMP-2 (WB)
Wu 2023 (China) [84]	In vivo	Hepatocellular carcinoma	Orthotopic Hepa1-6 liver cancer mouse model	*n* = 6 per group	1. Tumor burden (liver tumor area/photographs); 2. Angiogenesis markers CD31 and α-SMA (IF); 3. Liver index; 4. Serum inflammatory cytokines IL-6, IL-1β, IL-17, TNF-α (ELISA); 5. p-Akt1/Akt1, p-mTOR/mTOR, p-STAT3/STAT3, HIF-1α (WB); 6. HIF-1α (IHC)
Yan 2017 (China) [85]	In vitro	Colorectal cancer	SW620, HT-29, HCT116, HCT-8 human CRC cell lines	NR	1. Cell viability (MTT); 2. Colony formation; 3. Proliferation (CFDA-SE); 4. Apoptosis (Annexin V/PI FACS); 5. mRNA/protein of Survivin, PCNA, Cyclin D1, CDK4, Bcl-2, Bax; 6. Phosphorylation of AKT and ERK (Western blot)
Yang 2019 (China) [86]	In vitro + In vivo	Breast cancer	MDA-MB-231 and MDA- MB-453 TNBC cell lines + zebrafish xenograft	NR	1. Cell viability; 2. Colony formation; 3. Apoptosis (Hoechst, Annexin-V/PI); 4. Migration (wound healing assay); 5. Invasion (Transwell); 6. Protein levels: MMP2, MMP9, N-cadherin, Vimentin, CAV1; 7. In vivo metastasis in zebrafish
Yang 2025 (China) [12]	In vitro + In vivo	Cervical cancer	SiHa and CaSki human cervical cancer cell lines + BALB/c xenograft	In vitro: NR; In vivo: *n* = 5 per group	1. Cell viability (CCK-8); 2. Colony formation; 3. Migration (wound healing); 4. Apoptosis (Annexin V/PI); 5. Cell cycle (flow cytometry); 6. Western blot: BAX, BCL2, caspase-3, cleaved-caspase-3, CDK2, cyclin A, IL-17A, p-NF-κB p65; 7. Tumor volume & weight (xenograft); 8. Body weight toxicity assessment
Yao 2025 (China) [87]	In vitro	Lung cancer	H1299 human lung cancer cells + L929, A549, HepG2 for toxicity comparison	NR	1. Cell viability (MTT assay); 2. Live/dead staining (Calcein-AM/PI); 3. Confocal uptake imaging; 4. Western blot: PI3K, MTOR, HIF1A, VEGFA, PTEN; 5. qPCR: HIF1A, VEGFA, PTEN; 6. Hemolysis assay
Yuan 2024 (China) [88]	In vitro	Colorectal cancer	SW620 colorectal cancer cells + NCM460 normal colon cells	NR	1. Cell viability (MTS assay); 2. Colony formation assay; 3. Apoptosis assay (Annexin V/PI flow cytometry)
Zhang 2016 (China) [89]	In vitro	Ovarian cancer	A2780 ovarian cancer cells + IOSE80 normal ovarian epithelial cells	NR	1. Cell viability (MTT assay); 2. Apoptosis (DAPI staining; Annexin V/PI FACS); 3. Apoptosis-associated proteins (Western blot: cleaved caspase-3/9, Bcl-2); 4. Migration (wound healing assay; Transwell assay); 5. MMP expression (Western blot: MMP-2, MMP-9)
Zheng 2025 (China) [17]	In vitro	Hepatocellular carcinoma	Hepatocellular carcinoma cells	NR	1. Cell viability (MTT assay); 2. Apoptosis (flow cytometry Annexin V/PI); 3. IL6 and TNF mRNA expression (RT-qPCR); 4. IL6 and TNF protein expression (Western blot); 5. AGE-RAGE pathway protein expression (Western blot)
Zhou 2025 (China) [90]	In vitro + In vivo	Multiple cancers	A549, SGC7901, HCT116, PANC1, MDA-MB-231, CTC-TJH-01 cells + LLC hematogenous lung metastasis mouse model	In vitro: *n* = 3–12; In vivo: *n* = 4–9 per group	1. Cell viability (CCK-8, pre-adhesion vs. adhesion); 2. Tumor cell adhesion to ECM, ECs, platelets; 3. CTC proliferation and adhesion; 4. Phosphorylation of FAK (Tyr397) and Src (Tyr416); 5. In vivo lung metastatic nodules and foci; 6. In vivo TC-platelet and TC-EC interactions; 7. Body weight/toxicity
Zhu 2016 (China) [91]	In vitro + In vivo	Gastric cancer	SGC-7901 human gastric cancer cells + BALB/c nude mouse xenograft	In vitro: NR; In vivo: *n* = 10 per group	1. Cell viability (MTT assay); 2. Apoptosis (Annexin V-FITC/PI flow cytome- try); 3. Western blot: Mcl-1, Bcl-xl, Bcl-2, Bax, Bak, Bad, cytochrome c, caspase-3, caspase-9; 4. Tumor volume and mouse body weight (xenograft); 5. Apoptosis-related protein expression in tumor tissue (Western blot)
Zhu 2025 (China) [92]	In vitro	Colorectal cancer	Human colorectal cancer cell lines SW480 and HCT116	NR	1. Cell viability (CCK-8); 2. Colony formation; 3. EdU proliferation assay; 4. Apoptosis (flow cytometry); 5. Wound healing migration; 6. Transwell invasion; 7. RPLP1 mRNA expression (qPCR); 8. Proteomic profiling (TMT analysis).

AGE-RAGE, advanced glycation end products-receptor for AGE axis; AMPK, AMP-activated protein kinase; EMT, epithelial-mesenchymal transition; FAK, focal adhesion kinase; FOD, total flavonoids of *Oldenlandia diffusa*; HIF-1α, hypoxia-inducible factor-1α; MAPK, mitogen-activated protein kinase; MMP, matrix metalloproteinase; NR, not reported; STAT3, signal transducer and activator of transcription 3; UA, ursolic acid; uPA, urokinase-type plasminogen activator; VEGF-C, vascular endothelial growth factor C; VEGFR3, vascular endothelial growth factor receptor 3.

## Data Availability

Data supporting the findings of this study are available upon reasonable request to the corresponding author.

## Supplementary Materials

The following supporting information can be downloaded at: https://www.mdpi.com/article/10.3390/cancers18040672/s1, Supplementary Materials S1: Search Strategies Applied Across Databases; Supplementary Materials S2: Preclinical evidence of the antitumor activity and growth inhibition of HDW. ALT/AST, liver enzymes; AR, androgen receptor; ATO, arsenic trioxide; BLCA, bladder cancer; CDK, cyclin-dependent kinase; CTC, circulating tumor cell; EGFR, epidermal growth factor receptor; EMT, epithelial-mesenchymal transition; ER stress, endoplasmic reticulum stress; EV, extracellular vesicle; FOD, total flavonoids of *Oldenlandia diffusa*; GPX4, glutathione peroxidase 4; HDI, *Hedyotis diffusa* injection; HDP, *Hedyotis diffusa* polysaccharide; HDW, *Hedyotis diffusa* Willd.; HMOX1, heme oxygenase-1; HR, homologous recombination; IAP, inhibitor of apoptosis protein; IC50, half maximal inhibitory concentration; LC3B, autophagy marker; MAPK, mitogen-activated protein kinase; MMP, matrix metalloproteinase; PI3K/Akt/mTOR, phosphoinositide 3-kinase/Akt/mechanistic target of rapamycin pathway; ROS, reactive oxygen species; STAT3, signal transducer and activator of transcription 3; TTH, total triterpenoids of HDW; TSI, tumor suppression index; UA, ursolic acid; Supplementary Materials S3: Preclinical evidence of immune and tumor microenvironment-related effects of HDW. AMPK, AMP-activated protein kinase; DC, dendritic cell; ECM, extracellular matrix; EGFR, epidermal growth factor receptor; EMT, epithelial-mesenchymal transition; FAK, focal adhesion kinase; GZMB, granzyme B; HDI, *Hedyotis diffusa* injection; HDP, *Hedyotis diffusa* polysaccharide; HDW, *Hedyotis diffusa* Willd.; HIF-1α, hypoxia-inducible factor-1α; IFN, interferon; IL, interleukin; MMP/TIMP, matrix metalloproteinase and tissue inhibitor; NF-κB, nuclear factor kappa B; NK, natural killer cell; PI3K/Akt/mTOR, phosphoinositide 3-kinase/Akt/mechanistic target of rapamycin pathway; ROS, reactive oxygen species; STAT3, signal transducer and activator of transcription 3; TME, tumor microenvironment; TNF, tumor necrosis factor; Treg, regulatory T cell; VEGF/VEGFR, vascular endothelial growth factor and receptor.
