# Supplementary material for: Immune and Tumor Microenvironment Mechanisms of Hedyotis diffusa Willd: A Scoping Review and Network Pharmacology Analysis
Source: Cancers (Basel). 2026 Feb 18;18(4):672. doi: 10.3390/cancers18040672 (PMC12939048; doi:10.3390/cancers18040672)
Supplement: Supplementary file 1 [file cancers-18-00672-s001.zip › cancers-4116145-supplementary.pdf]

SUPPLEMENTARY

Supplementary Materials S1. Search Strategies Applied Across Databases

Database	Search Strategy	Records Retrieved
**PubMed**	((cancer*) OR (carcinoma*) OR (neoplasm*) OR (tumor*)) AND ((Hedyotis diffusa*) OR (Oldenlandia diffusa*) OR (Baihuasheshecao*))	**227**
**EMBASE**	('cancer'/exp OR cancer*:ab,ti OR carcinoma*:ab,ti OR neoplasm*:ab,ti OR tumor*:ab,ti) AND ((('hedyotis diffusa'/exp OR 'oldenlandia diffusa'/exp OR hedyotis) AND diffusa*:ab,ti OR oldenlandia) AND diffusa*:ab,ti OR baihuasheshecao*:ab,ti)	**301**
**CENTRAL**	(cancer* OR carcinoma* OR tumor*) AND ("Hedyotis diffusa" OR "Oldenlandia diffusa" OR Baihuasheshecao)	**16**
**OASIS**	“백화사설초”	**22**

Supplementary Materials S2. Preclinical evidence of the antitumor activity and growth inhibition of HDW

Study ID	Intervention	Dose / Exposure conditions	Comparator	Main findings of the study
**Bai 2025a[1]**	HDW injection	In vitro dose: NR; In vivo: HDW injection for 2 weeks	Vehicle control	1. GPX4↓, SLC7A11↓, FTH1↓, COX2↑, TFRC↑, HMOX1↑ indicating ferroptosis; 2. Tumor volume↓ and tumor weight↓ vs control ($p < 0.05$ to $p < 0.0001$); 3. Ki67+ proliferation index ↓ ($p < 0.01$); 5. HDW induces ferroptosis-mediated antitumor activity in BLCA.
**Chen 2018[2]**	Ethanol extract of HDW (EEHDW)	0.25, 0.5, 1, 2 mg/mL EEHDW for 24–48 h; TGF-β1 at 5 ng/mL used to induce EMT	Control (no TGF-β) and TGF-β group	1. TGF-β ↑ viability; EEHDW ↓ viability dose-dependently; 2. TGF-β ↑ cell density; EEHDW reduced density.
**Chen 2022[3]**	Total flavonoids of Oldenlandia diffusa (FOD)	Cell experiments: 12.5, 20, 25 µg/mL for 24–48h; Animal: 0.4 mg/kg/day i.p. for 2 weeks	Model group (saline)	1. FOD ↓ proliferation dose- & time-dependently; 2. FOD ↓ Ki67; 3. FOD induced G0/G1 arrest; 4. FOD ↑ apoptosis; 5. FOD ↑ cleaved caspase-3; 6. FOD ↑ LC3B-II & P62; 7. FOD ↑ autophagic flux; 8. FOD ↑ ER-stress signaling; 9. FOD ↑ ROS; 10. FOD ↓ tumor size & weight; 12. No major toxicity (ALT/AST/UREA/CREA unchanged).
**Chen 2025[4]**	Quercetin treatment	10–320 µM quercetin for 24, 48, 72 h (MTT); 40–80 µM for 48 h (RT-qPCR, WB)	Vehicle control (0 µM, DMSO)	1. Quercetin ↓ viability in MCF-7 (dose/time-dependent); 2. Quercetin ↓ BIRC5 mRNA; 3. Quercetin ↓ CDK1 mRNA; 4. Quercetin ↓ FOS mRNA; 5. Quercetin ↓ HSP90AA1 mRNA; 6. Quercetin ↓ BIRC5 protein; 7. Quercetin ↓ CDK1 protein; 8. Quercetin ↓ HSP91α (FOS protein unchanged).
**Cheng 2022[5]**	HDW aqueous extract	Mice: HDW 100 mg/kg/day orally for 8–12 weeks; Cell model: HDW extract 100–400 µg/mL for 24–48 h	HFD model group without HDW	4. HDW ↓ tumor incidence and ↓ tumor size; 8. HDW ↓ Ki67+ cells and ↓ α-SMA (reduced fibrosis).
**Cheng 2025[6]**	HDW-derived extracellular vesicle–like particles (HDW-EVLPs)	Particle-to-cell ratios: 10^3, 10^4, 10^5; exposure 24–48 h	Blank control (no EV treatment)	1. HDW-EVLPs internalized within 4 h; 2. HDW-EVLPs ↓ viability dose- & time-dependently; 3. Minimal effect on normal WRL68 viability; 4. Huh-7 showed shrinkage & pseudopodia loss; 5. HDW-EVLPs ↑ apoptosis; 6. Cell cycle arrest (G0/G1↓, S-phase↑); 7. DEGs enriched in PI3K/Akt & apoptosis pathways; 8. qRT-PCR: PI3K/AKT/mTOR/Bcl-2↓, p53/Bax/Caspases↑; 9. Western blot confirmed ↑ p54 & caspases.
**Chung 2017[7]**	OD aqueous extract (Oldenlandia diffusa)	In vitro: 100–500 µg/mL for 24–48 h; In vivo: 100 mg/kg/day oral gavage for 21 days	Vehicle control (PBS or medium)	1. OD ↓ viability dose- and time-dependently; 2. OD ↑ apoptosis; 3. OD ↑ ROS; 4. OD ↓ mitochondrial membrane potential; 5. OD ↑ Bax, cytochrome c, caspase-3/9 and ↓ Bcl-2; 6. OD ↓ tumor volume & weight; 8. OD inhibits CRC via ROS-mediated mitochondrial apoptosis.
**Feng 2017[8]**	EEHDW	In vitro: 0–2 mg/mL for 24–48 h; In vivo: 1 g/kg/day oral gavage for 16 days	Vehicle control	1. EEHDW ↓ cell viability; 2. ↓ tumor volume & weight; 3. ↓ Ki-67; 4. ↑ TUNEL; 5. ↑ cytochrome c, caspase-3/9, PARP; 6. ↓ Pim-1 & Bcl-2 and ↑ Bax.
**Feng 2025[9]**	TFHDW (Total Flavonoids of HDW)	Cell lines: 10–160 µM (48 h); Functional assays at 40 µM (RM1) and 30 µM (LNCaP);	Blank/vehicle; si-PIAS4; oe-	1. TFHDW ↓ viability (IC50 39.56 µM, 29.78 µM); 2. ↓ colony formation; 3. ↑ apoptosis; 4. ↑ TUNEL; 5. ↓ migration/invasion; 9. TFHDW ↑ DDB2

		Mice: 50 mg/kg/day oral gavage × 3 weeks	STAT3; MG-132; sh-PIAS4	transcription & ↑ AR ubiquitination; 10. In vivo: ↓ tumor size, ↓ Ki67, ↑ apoptosis, ↑ AR ubiquitination.
**Han 2023[10]**	EEOD (Ethanol Extract of Oldenlandia diffusa)	2D assays: 0–8 mg/mL for 17–24 h; 3D invasion assay: 0, 1, 2, 3 mg/mL continuously perfused (1 μL/min) for 24 h	Control (0 mg/mL EEOD); Positive control: temozolomide 100 μmol/L	1. EEOD ↓ viability of U87/U251 dose-dependently (IC50 U87 = 4.106 mg/mL; U251 = 2.725 mg/mL); 2. EEOD ↑ apoptosis in U87 dose-dependently; 6. Network analysis predicted targets including MAPK, EGFR, MYC, GSK3B; 7. Mechanism: inhibition of MAPK/Wnt signaling and regulation of microtubule cytoskeleton.
**Ho 2018[11]**	HDW extract in DMSO	24–48 h treatment; concentrations 0–4000 μg/mL; IC50 (HCC2998) = 2800→1000 μg/mL; IC50 (KM12) not reached	Vehicle (1% DMSO)	1. HD ↓ viability of HCC2998 dose- & time-dependently (IC50 2800→1000 μg/mL); weak effect on KM12 (IC50 not reached); 2. HD ↓ migration in HCC2998 (IC50 700 μg/mL) and KM12 (IC50 920 μg/mL); 3. HD ↓ colony number dose-dependently in both cell lines; 5. HD ↓ colony area dose-dependently in both cell lines.
**Huang 2021[12]**	HDW crude extract	In vitro: 0–2 mg/mL (24–72 h); In vivo: 100 mg/kg/day × 14 days (oral)	Vehicle control	1. HDW ↓ cell viability dose-dependently; 2. HDW ↓ colony formation; 3. HDW ↓ migration in all 3 HCC lines; 4. HDW ↓ phosphorylation of AKT, mTOR, ERK, 4EBP1; 5. HDW ↓ Bcl-2 and ↑ Bax (pro-apoptotic shift); 6. HDW ↓ tumor volume & weight in vivo; 7. No significant body-weight loss; 8. HDW ↓ Ki67+ proliferating cells; 9. No evident liver/kidney toxicity in H&E.
**Huang 2022[13]**	Hedyotis diffusa Injection (HDI)	In vitro: 0–100 μg/mL, 24–48h; In vivo: 15 mg/kg/day IM × 21d	NS control, cisplatin	1. HDI ↓ viability in A549/H1975; 2. HDI ↓ colony formation; 3. HDI ↓ migration & invasion; 10. HDI ↓ tumor volume & weight; 11. No major toxicity on organ histology.
**Jiang 2017[14]**	Total coumarins of HDW (TCHD)	0–125 μg/mL for 24–48 h; IC50 ≈ 100–104 μg/mL	Untreated control (0.05% DMSO)	1. TCHD ↓ viability dose-dependently; 2. TCHD ↑ apoptotic morphology; 3. TCHD ↑ Annexin V+ apoptosis; 4. TCHD ↑ caspase-3/8/9 activation and ↑ PARP cleavage; 5. TCHD ↓ PI3K, Akt, p-Akt, p-P65 (PI4K/Akt/NF-κB inhibition)
**Jing 2023[15]**	2-hydroxy-3-methylanthraquinone (HMA)	0–200 μmol/L for 24–48 h (in vitro); 100 μmol/L HMA (in vivo, duration NR)	Vehicle control	1. HMA ↓ viability (dose-dependent); 2. HMA ↓ colony formation; 3. HMA ↑ apoptosis (↑ Annexin V+, ↑ cleaved apoptotic proteins); 4. HMA ↓ migration and invasion; 5. HMA ↓ tumor mass and volume in vivo; 6. HMA ↓ Ki-67 expression; 10. HMA ↑ γ-H2A.X nuclear localization (↑ DNA damage); 11. HMA sensitized cells to RI-1 and AZD7762, indicating HR repair inhibition; 12. MYC overexpression rescued, whereas si-MYC enhanced HMA effects; AKT inhibition (MK2206) enhanced HMA → confirming PI3K–AKT–MYC–CHK1–RAD51 axis involvement.
**Kim 2018[16]**	Ursolic acid (UA)	0–80 μM for 24 h	Vehicle control (0 μM)	1. UA ↓ viability dose-dependently; 2. UA ↑ TUNEL+ apoptotic cells; 3. UA ↑ sub-G1 apoptotic fraction; 4. UA ↑ cleaved PARP and cleaved caspase-3.
**Kuo 2017[17]**	HDW ethanol extract ± ATO	In vitro: HDW 0.1–1.6 mg/mL, ATO 0.8–12.8 μM for 24–48 h; In vivo: HDW 100/250/500 mg/kg/day PO × 2 weeks; ATO 5 mg/kg/day	Vehicle control; ATO alone; HDW alone	1. HDW ↓ viability dose- & time-dependently; 2. HDW ↑ apoptosis (↑ cleaved PARP, caspase-3/-8/-9); 3. HDW ↑ DR4/DR5 expression; 4. HDW ↓ Bcl-2, Bcl-xL, survivin and ↑ Bak, t-Bid; 5. In vivo: HDW ↓ spleen enlargement (dose-dependent) with no liver toxicity; 6. HDW ↑ survival of leukemic mice; 7. HDW×ATO combination synergistically ↑ cytotoxicity, ↑ apoptosis, ↑ DR4/DR5 activation, ↑ caspase cascade, ↓ anti-apoptotic proteins vs single agents.

**Lai 2017[18]**	HDW ethanol extract (EEHDW)	0–2.0 mg/mL EEHDW for 12–48 h	Vehicle control	1. EEHDW ↓ viability in a dose- & time-dependent manner.
**Lee 2016[19]**	Ethanol extract of OD (EOD)	50–400 μg/mL; 24–72 h	untreated control	1. Viability ↓ dose-dependently; 2. Sub-G1 population ↑ (apoptosis ↑); 3. Mitochondrial depolarization ↑; 4. Caspase-3/-9 activities ↑ and inhibited by zVAD-fmk; 5. ROS ↑ and NAC abolished cytotoxicity; 7. EOD enhanced chemosensitivity to all tested agents
**Lee 2019[20]**	Intracellular extract of OD	0–400 μg/mL for 24–48 h (in vitro); OD administered for 4 weeks (dose NR)	Vehicle control	1. OD did not significantly affect viability at ≤200 μg/mL; 2. OD ↓ migration; 3. OD ↓ invasion; 6. OD ↓ lung metastatic nodules, confirming anti-metastatic activity in vivo.
**Li 2016[21]**	HDW ethyl acetate fraction (H-EtOAc) ± DMQ	Extract: 62.5–500 μg/mL for 24 h; DMQ: 62.5–500 μmol/L for 24 h	Vehicle control	1. H-EtOAc and DMQ ↓ viability dose-dependently; 2. Apoptosis ↑ (AO/EB, Annexin V/PI, DNA ladder); 3. Caspase-3/8/9 ↑ indicating mitochondrial & death-receptor apoptosis; 4. MMP ↓ and ROS ↑; 5. p53, Bax, Fas, FasL, p21 ↑; Bcl-2, cyclin E, CDK2 ↓; 6. Cytochrome C release ↑ (mitochondrial→cytosolic); 7. mRNA: Bax↑, p53↑, Bcl-2↓; 8. G0/G1 arrest; overall DMQ mainly activates mitochondrial apoptosis, H-EtOAc mainly activates death-receptor apoptosis
**Li 2017[22]**	Hedyotis diffusa extract (HHD extract)	50, 100, 200 mg/L for 24–72 h	Vehicle control	1. HHD extract ↑ apoptotic morphology (chromatin condensation, apoptotic bodies); 2. HHD extract ↑ apoptosis rate dose- and time-dependently ($p<0.05$); 3. HHD extract ↓ survivin expression (dose-dependent, $p<0.01$); 4. HHD extract ↓ livin expression (dose-dependent, $p<0.02$), indicating apoptosis induction via downregulation of IAP family proteins
**Li 2018[23]**	EEHDW	0.5–2.0 mg/mL for 24–48 h	Vehicle control (0.1% DMSO)	1. EEHDW ↓ viability dose/time-dependently; 2. EEHDW ↓ colony formation; 3. EEHDW ↑ apoptosis (Annexin V/PI, nuclear condensation); 4. EEHDW ↓ Bcl-2, ↓ Cyclin D1, ↓ CDK4 mRNA & ↑ Bax, ↑ p21; 5. EEHDW ↓ Bcl-2, ↓ Cyclin D1, ↓ CDK4 proteins & ↑ Bax, ↑ p21; 6. EEHDW ↑ PTEN and ↓ PI3K, ↓ p-AKT indicating inhibition of PI3K/AKT-driven drug resistance
**Lin 2019b[24]**	Hedyotis diffusa polysaccharide (HDP)	25, 100, 200 μg/mL for 48 h (in vitro); 50 or 100 mg/kg/day PO × 15 days (in vivo)	Vehicle control (PBS); Cisplatin 4 mg/kg as positive control	1. HDP ↓ A549 viability dose-dependently but no cytotoxicity in WI38; 2. HDP ↓ colony formation; 3. HDP ↑ apoptosis from 2.1% → 28.85–66.08%; 4. HDP ↑ caspase-9/3 activation (≥2-fold); 5. HDP ↑ cytochrome c release to cytosol; 6. HDP ↑ Bax with unchanged Bcl-2; 7. HDP ↑ Bax/Bcl-2 ratio; 8. HDP ↓ tumor growth significantly at 50 and 100 mg/kg; 9. HDP ↓ tumor weight with inhibitory effect comparable to cisplatin, without toxicity
**Ling 2023[25]**	Oldenlandia diffusa total flavonoids	64, 96, 128, 160 μg/mL for 24–72 h (in vitro); 50, 100, 200 mg/kg every 2 days ×10 doses (in vivo)	Vehicle control (RPMI-1640 or saline)	1. Flavonoids ↓ viability of MKN-45/AGS in a dose- and time-dependent manner; 2. Flavonoids ↑ apoptosis (early + late) with chromatin condensation; 3. Flavonoids induced G1/S arrest via ↓ Cyclin A and ↓ Cdk2; 4. Flavonoids ↑ ROS (blocked by NAC, enhanced by H2O2); 5. Flavonoids ↓ MMP (JC-1 red/green ratio ↓); 6. Flavonoids ↑ Bax, ↑ Apaf-1, ↑ Cleaved-caspase-3 and ↓ Bcl-2, ↓ Pro-caspase-9, ↓ mitochondrial Cytochrome C indicating mitochondrial apoptosis activation; 7. Flavonoids ↓ tumor volume and weight in mice; 8. Flavonoids ↓ PCNA and Ki-67 in tumor tissues; 9. Flavonoids ↓ serum CA72-4 levels

**Lu 2016[26]**	Oldenlandia diffusa aqueous extract (ODE)	10–200 μg/mL for 24–96 h (in vitro); 0.2 or 1.0 g/kg i.p. daily × 30 days (in vivo)	Vehicle (saline)	1. ODE ↓ viability in CRC cells dose-/time-dependently; 2. ODE ↓ colony formation and ↓ BrdU incorporation; 3. ODE ↑ cell death; 4. ODE ↑ apoptosis (caspase-3, histone-DNA, Annexin V, TUNEL); 5. ODE ↑ cleaved PARP and ↑ cleaved caspase-3; 6. ODE ↑ AMPK activation (↑ p-AMPK, ↑ p-ACC); 7. ODE ↓ mTORC1 activation (↓ p-S6K1) and ↓ Bcl-2/HIF-1α; 8. ODE ↑ AMPK–p53 association and ↑ p53 (p-Ser15); 9. AMPK or p53 knockdown attenuated ODE-induced apoptosis and growth inhibition; 10. In vivo ODE ↓ tumor volume, ↓ tumor weight, ↓ tumor growth without toxicity; 11. Xenograft tumors showed ↑ AMPK activation, ↑ p53 activation, and ↓ mTORC1 signaling
**Lv 2021[27]**	HDW-isolated alternariol derivatives	NR (dose–response for IC50 assessment over 48 h)	Vehicle control (DMSO)	1. Compounds 1 and 2 showed strong cytotoxicity on A2780 cells (IC50 = 3.1 μM and 9.4 μM); 2. Compounds 1 and 2 more cytotoxic than alternariol (parent compound); 3. p-coumaroyl substituent > caffeoyl substituent for potency; 4. Compound 7 showed weak cytotoxicity (HepG2 IC50 = 76.5 μM; A549 IC50 = 65.2 μM)
**Ma 2022[28]**	HDW ethanol extract ± ursolic acid	UA: 0–100 μM (48 h) for cytotoxicity; 5–15 μM (48 h) for gene expression; UA 40 μM for colony assay; In vivo UA 20 mg/kg IP 3×/week for 4 weeks; HDW extract 0–1000 μg/mL (48 h)	vehicle; 5-FU; estradiol	1. ↓ viability dose-dependently; 2. ↓ colony formation; 3. ↓ CYP19A1 mRNA; 4. ↓ Ar protein; 5. UA synergized with 5-FU (CI 0.70–0.77); 6. ↓ tumor volume with UA (~90% TSI); 7. ↓ tumor weight
**Ning 2022[29]**	Hedyotis diffusa water extract	0–8 mg/mL for 24–48 h (in vitro); oral HDW 200 mg/kg/day for 21 days (in vivo)	Vehicle control	1. HDW ↓ viability and ↓ colony formation; 2. HDW ↑ apoptosis (↑ Bax, ↑ cleaved caspase-3/PARP, ↓ Bcl-2); 3. HDW induced G0/G1 arrest; 4. HDW ↓ migration and ↓ invasion; 5. HDW ↑ ROS and ↓ mitochondrial membrane potential; 6. HDW ↓ p-Akt and ↓ p-ERK; 7. In vivo: HDW ↓ tumor volume and ↓ tumor weight; 8. HDW ↓ Ki-67 and ↑ TUNEL+ apoptotic cells, indicating anti-proliferative and pro-apoptotic effects
**Ou 2024[30]**	Hedyotis diffusa water extract (repeat)	0–8 mg/mL for 24–48 h (in vitro); oral HDW 200 mg/kg/day × 21 days (in vivo)	Vehicle control	1. HDW ↓ viability and ↓ colony formation; 2. HDW ↑ apoptosis (↑ Bax, ↑ cleaved caspase-3/PARP, ↓ Bcl-2); 3. HDW ↑ ROS and ↓ mitochondrial membrane potential; 4. HDW ↓ migration and ↓ invasion; 5. HDW ↓ p-Akt and ↓ p-ERK; 6. In vivo: HDW ↓ tumor volume and ↓ tumor weight; 7. HDW ↓ Ki-67 and ↑ TUNEL+ apoptotic cells, indicating anti-proliferative and pro-apoptotic activity via ROS–mitochondrial–Akt/ERK pathways
**Pu 2016[31]**	HDW water extract ± cisplatin	HDW 0–200 μL/mL; cisplatin 0–8.9 μg/mL; combination: HDW 140 μL/mL + cisplatin 6 μg/mL for 24–48 h	Vehicle control; cisplatin alone; HDW alone	1. HDW ↓ viability dose- & time-dependently; 2. HDW caused G2/M arrest; 3. HDW ↑ apoptosis; 4. HDW ↓ migration; 5. HDW ↓ invasion; 6. HDW ↑ Bax/Bad and ↓ Bcl-xl/Bcl-2; 7. HDW ↑ active caspase-3, ↑ PARP cleavage, ↑ active caspase-8; 8. Combination HDW+cisplatin showed stronger inhibition, higher apoptosis, and stronger caspase activation than either agent alone
**Sun 2016[32]**	EEHDW (ethanol extract of HDW)	0, 0.5, 1, 2 mg/mL for 24 h	Vehicle control	1. EEHDW ↓ SP proportion dose-dependently; 2. EEHDW ↓ Lgr5 protein expression; 3. EEHDW ↓ sphere formation (>50-cell spheroids) in a dose-dependent manner; 4. EEHDW ↓ viability of SP cells; 5. EEHDW induced cell shrinkage and loss of confluence; 6. EEHDW ↓ ABCB1, ↓ β-catenin, ↓ c-Myc,

				↓ PCNA, ↓ survivin mRNA levels, indicating inhibition of CSC features and Wnt/ABC transporter pathways
**Trang 2025[33]**	Hedyotis diffusa essential oils	5–100 μg/mL for 48 h (MTT assay)	Vehicle control (DMSO); positive control: ellipticine	1. Essential oil cytotoxicity: MCF-7 IC50 = 10.99–23.14 μg/mL; SK-LU-1 IC50 = 10.86–16.69 μg/mL; HepG2 IC50 = 14.92–18.02 μg/mL; 2. Antioxidant activity moderate (IC50 = 243.78–300.54 μg/mL); 3. NO inhibition strong (IC50 = 36.81–40.58 μg/mL); 4. α-Glucosidase inhibition weak (IC50 ≈ 459.96–475.37 μg/mL); 5. Antimicrobial activity strongest against B. subtilis (MIC 32–64 μg/mL).
**Wang 2017[34]**	HDW-derived isolated compounds	MTT assay; compounds tested 1–300 μM; IC50 values calculated	Vehicle control (0 μM)	1. Compound 3 showed strong cytotoxicity except HeLa (IC50 = 9.6–62.2 μM); 2. Compound 4 showed moderate cytotoxicity across all lines (IC50 = 33.6–89.3 μM); 3. Compounds 1 and 9 moderate cytotoxicity to HCT15, A459, HepG2; 4. Compound 7 moderate cytotoxicity to HCT15 and A459; 5. Other compounds largely inactive (IC50 > 100 μM).
**Wang 2018a[35]**	Pure isolated compounds from Hedyotis diffusa	MTT cytotoxicity assay; IC50 measured after compound exposure (μM)	Vehicle control (0 μM)	1. Compound 1 showed strong cytotoxicity across all tested lines (IC50 9.5–28.2 μM); 2. Compound 2 strong cytotoxicity to 7/8 lines (IC50 15.8–26.2 μM); 3. Compound 11 strong activity to HL-60, A549, HepG2, BGC-823, CNE-2, HCT15 (IC50 16.5–40.4 μM); 4. Compounds 4–7 showed moderate cytotoxicity (IC50 26.2–49.4 μM); 5. Compounds 12–13 showed moderate cytotoxicity to several lines (IC50 26.2–48.3 μM); 6. Compounds 8–10 showed selective but weaker cytotoxicity (>50 μM in many lines).
**Wang 2021b[36]**	OD extract ± isolated UA	In vitro doses: NR; In vivo dose: NR	Vehicle control	1. UA ↓ viability of breast cancer cells; 2. UA ↓ migration and ↓ invasion; 6. UA ↓ tumor growth and ↓ metastasis in vivo.
**Wang 2021a[37]**	Hedyotis diffusa ethyl acetate extract (HDE)	0–200 μg/mL HDE for 48 h (CCK-8); 171.7 nmol/L BIIB021; 24 h for telomerase, apoptosis, WB	Vehicle control; BIIB021 single treatment; hTERT-siRNA	1. HDE inhibited SKM-1 proliferation (IC50 = 98.03 μg/mL); 2. HDE ↓ telomerase activity (p<0.01); 3. HDE ↓ HSP90 expression (p<0.01); 4. HDE ↑ apoptosis (p<0.01); 5. HDE ↑ cleaved caspase-3, cleaved caspase-8, cleaved PARP; combination of HDE + BIIB021 showed synergistic apoptosis-inducing effects; hTERT knockdown further enhanced all antitumor effects.
**Wang 2023[38]**	Kaempferol treatment	0–80 μM for 24–48 h in vitro; 20 or 60 mg/kg/day i.p. in vivo for tumor-bearing nude mice	Vehicle control; Cisplatin (positive control in vitro/in vivo)	1. Kaempferol ↓ viability of A549/H1299 cells dose- & time-dependently; 2. EdU incorporation↓ indicating proliferation suppression; 3. Apoptosis↑ in a dose-dependent manner; 4. Autophagosomes & autolysosomes↑ (confocal) and TEM double-membrane structures↑; 5. LC3B & Beclin-1↑, p62↓ supporting increased autophagic flux; 8. Overexpression of MET reversed kaempferol-induced autophagy and viability loss; 9. Autophagy inhibitors (3-MA, autophinib, si-Beclin1) reversed LC3B↑, p62↓, apoptosis↑; 10. In vivo: tumor weight↓, volume↓, fluorescence↓; 11. IHC confirmed ↑autophagy markers and ↓MET/PI3K/AKT/mTOR signaling in tumors.
**Wang 2024[39]**	Hedyotis diffusa extract	0, 50, 75, 100, 125, 150 μg/mL for 48 h	Control (0 μg/mL)	1. Cell viability↓ dose-dependently (IC50 ≈ 79.65 μg/mL); 2. Apoptosis↑ (Control 9.05% → Low 32.53% → High 74.43%); 3. CTNNB1 protein↓ vs control (p<0.05), high dose↓ > low dose↓; 4. CTNNB1 mRNA↓ in dose-dependent manner (p<0.05)

**Wu 2017[40]**	Hedyotis diffusa polysaccharides (HDP)	25–800 μg/mL for 24–72 h	Control (untreated)	1. Cell viability↓ in dose- and time-dependent manner; 2. G0/G1 arrest↑ (21.2%→68.4%); 3. Apoptosis↑ (23.44%→67.45% across 100–800 μg/mL); 4. Caspase-3/8/9 activation↑; 5. Bcl-2↓; 6. Cell invasion↓ (54.33%~72.21%); 7. MMP-2↓ and uPA↓ indicating inhibition of invasive phenotype.
**Wu 2022[41]**	Total triterpenoids of Hedyotis diffusa (TTH)	1–75 μg/mL for 24–48 h	Vehicle control	1. Cell viability ↓ dose- and time-dependently; 2. G0/G1 arrest ↑; 3. Ki67 expression ↓; 4. Apoptosis ↑; 5. Bax ↑, Bcl-2 ↓, cleaved Caspase-3 ↑; 6. Migration ↓; 7. MMP-2 ↓, MMP-9 ↓, TIMP-2 ↑ indicating inhibited metastatic phenotype.
**Wu 2023[42]**	HDW injection	50, 100, 150 mg/kg i.p., every other day for 14 injections	Vehicle control	1. Tumor burden↓ dose-dependently.
**Yan 2017[43]**	Chloroform extract of Hedyotis diffusa Willd (CEHDW)	12.5–100 μg/mL for 24 h (SW620 dose-response); 0–500 μg/mL for 24–48 h (fraction screening)	Vehicle control (DMSO <0.5%)	1. Viability↓ across CRC lines; CEHDW most potent; 2. Colony number↓; 3. Proliferation↓ (fluorescence shift↑); 4. Apoptosis↑ dose-dependently (SW620 early+late apoptosis ~6.6%→~79.5% from 0→100 μg/mL); 5. Survivin↓, PCNA↓, Cyclin D1↓, CDK4↓, Bcl-2↓, Bax↑; 6. p-AKT↓ and p-ERK↓ indicating blockade of AKT/ERK signaling and pro-apoptotic/anti-proliferative mechanism.
**Yang 2019[44]**	Ethanol extract of Oldenlandia diffusa (EEOD)	20–50 μg/mL for 24–48 h (in vitro); 20–40 μg/mL in water (zebrafish model)	Vehicle control	1. ↓ proliferation dose-dependently; 2. ↓ colony formation; 3. ↑ apoptosis and ↑ p-H2AX; 4. ↓ wound closure rate; 5. ↓ invasion counts.
**Yang 2025[45]**	HDW 20:1 concentrated extract	2–6 mg/mL for 24–48 h (in vitro); 600 mg/kg by gavage daily × 21 days (in vivo)	Vehicle control (0 mg/mL or saline)	1. ↓ viability dose-dependently (IC50 SiHa 2.616–2.773 mg/mL); 2. ↓ colony formation; 3. ↓ migration; 4. ↑ apoptosis with ↑BAX and ↑cleaved-caspase-3 and ↓BCL2/caspase-3; 5. S-phase arrest via ↓CDK2 and ↓cyclin A; 7. ↓ tumor growth in vivo; 8. No significant systemic toxicity.
**Yao 2025[46]**	Fisetin extracted from HDW	50–300 μg/mL for 24 h	Vehicle control (culture medium or PBS)	1. Cell viability↓ dose-dependently, IC50 ≈203 μg/mL; 2. Apoptotic cells↑ with increasing dose; 3. Increased intracellular uptake over time.
**Yuan 2024[47]**	β-sitosterol extracted from HDW	10–40 μM for 24–48 h	Vehicle control (DMSO <0.1%)	1. ↓ cell viability dose-dependently (IC50 ≈19.86 μM for SW620, no toxicity to NCM460 IC50 ≈69.99 μM); 2. ↓ colony formation significantly at 20–40 μM; 3. ↑ apoptosis rate in a concentration-dependent manner.
**Zhang 2016[48]**	HDW extract	50–800 μg/mL for 24–48 h	Vehicle control (medium/DMSO)	1. ↓ viability dose- and time-dependent; no toxicity to IOSE80; 2. ↑ apoptosis with nuclear condensation and increased Annexin V+/PI+ cells; 3. ↑ cleaved caspase-3/9 and ↓ Bcl-2; 4. ↓ migration rate in wound healing and Transwell assays; 5. ↓ MMP-2 and MMP-9 expression indicating reduced metastatic potential.
**Zheng 2025[49]**	Quercetin from Hedyotis diffusa	Multiple doses; dose-dependent effects reported	Vehicle control (untreated)	1. Quercetin ↓ proliferation in a dose-dependent manner; 2. Quercetin ↑ apoptosis dose-dependently.
**Zhou 2025[50]**	Hedyotis diffusa Willd. granules (water extract)	In vitro: 0–3 mg/mL for 0.5–48 h; In vivo: 150 or 300 mg/kg/day orally for 21 days	Vehicle control; FAK inhibitor (Y15 10 μM); Src inhibitor (PP2 10 μM)	1. HDW ↓ viability primarily at pre-adhesion stage vs adhesion; 2. HDW ↓ adhesion to ECM, ECs, and platelets in a time- and dose-dependent manner; 3. HDW ↓ CTC proliferation and ↓ CTC–platelet adhesion; 5. HDW ↓ lung metastatic burden and foci; 7. No significant body weight change.

**Zhu 2016[51]**	2,7-dihydroxy-3-methylanthraquinone (DDMN)	Cell experiment: 10, 20, 40 μM for 48 h; Animal: 40 mg/kg i.p. daily for 20 days	Vehicle (0.5% DMSO)	1. ↓ cell viability dose-dependently (IC50 ≈ 20.92 μM); 2. ↑ apoptosis significantly with increasing concentrations; 3. ↓ Bcl-2/Bcl-xl, ↑ Bax, ↑ Bad (at 40 μM), ↑ cleaved caspase-3 and caspase-9, ↑ cytosolic cytochrome c indicating mitochondrial apoptosis; 4. ↓ tumor volume significantly without affecting body weight; 5. Xenograft tumors showed same apoptotic protein changes confirming mechanism.
**Zhu 2025[52]**	Active compounds of HDW (mix)	UA, OA, quercetin 0–70 μM for 72 h for cytotoxicity in SW480 cells; UA 12 μM for 72 h for TMT-based proteomics in SW480 cells; UA 10–20 μM for 24–72 h for RPLP1 and functional assays	DMSO vehicle control; shCtrl/NC for shRNA groups; UA alone vs RPLP1 knockdown alone vs combination	1. UA dose-dependently ↓ cell viability; OA minimal effect; quercetin moderate effect; UA showed the lowest IC50 and strongest antiproliferative effect; 2. UA significantly ↓ colony formation; 3. UA ↓ EdU-positive proliferating cells; 4. UA ↑ apoptosis rate; 5. UA ↓ wound closure rate in migration assay; 6. UA ↓ invasion capacity in Transwell assay.

ALT/AST, liver enzymes; AR, androgen receptor; ATO, arsenic trioxide; BLCA, bladder cancer; CDK, cyclin-dependent kinase; CTC, circulating tumor cell; EGFR, epidermal growth factor receptor; EMT, epithelial–mesenchymal transition; ER stress, endoplasmic reticulum stress; EV, extracellular vesicle; FOD, total flavonoids of Oldenlandia diffusa; GPX4, glutathione peroxidase 4; HDI, Hedyotis diffusa injection; HDP, Hedyotis diffusa polysaccharide; HDW, Hedyotis diffusa Willd.; HMOX1, heme oxygenase-1; HR, homologous recombination; IAP, inhibitor of apoptosis protein; IC50, half maximal inhibitory concentration; LC3B, autophagy marker; MAPK, mitogen-activated protein kinase; MMP, matrix metalloproteinase; PI3K/Akt/mTOR, phosphoinositide 3-kinase/Akt/mechanistic target of rapamycin pathway; ROS, reactive oxygen species; STAT3, signal transducer and activator of transcription 3; TTH, total triterpenoids of HDW; TSI, tumor suppression index; UA, ursolic acid.

Supplementary Materials S3. Preclinical evidence of immune and tumor microenvironment–related effects of HDW

Study ID	Intervention	Dose / Exposure conditions	Comparator	Main findings of the study
**Bai 2025a[1]**	HDW injection	In vitro dose: NR; In vivo: HDW injection for 2 weeks	Vehicle control	4. CAV1↓, JUN↓, VEGFA↓ indicating TME modulation.
**Bai 2025b[53]**	Hedyotis diffusa polysaccharide (HDP) ± PD-1 + CTLA-4 antibodies	100 mg/kg HDP i.p. every 2 days; anti-PD-1 and anti-CTLA-4 10 mg/kg i.p. every 3 days	Vehicle (IgG control) and PD-1 + CTLA-4 treatment groups alone	1. HDP ↓ tumor volume and ↓ tumor weight ($p<0.05$–0.001); 2. HDP ↑ CD4+ and CD8+ infiltration; combination therapy ↑↑; 3. HDP ↑ GZMB, TNFα, IFN-γ in tumors; 4. HDP ↑ CD8+, GZMB+, TNFα+, IFN-γ+ T cells; 5. HDP minimal changes in Treg/B/NK/DC but ↓ tumor-associated macrophages.
**Chen 2018[2]**	Ethanol extract of HDW (EEHDW)	0.25, 0.5, 1, 2 mg/mL EEHDW for 24–48 h; TGF-β1 at 5 ng/mL used to induce EMT	Control (no TGF-β) and TGF-β group	3. EEHDW ↓ migration (84.1%→5.67%); 4. EEHDW ↓ invasion (60.3%→3.40%); 5. EEHDW reversed EMT (E-cadherin ↑; N-cadherin & Vimentin ↓); 6. EEHDW ↓ TGF-β, p-Smad2/3, Smad4 blocking EMT signaling.
**Chen 2022[3]**	Total flavonoids of Oldenlandia diffusa (FOD)	Cell experiments: 12.5, 20, 25 µg/mL for 24–48h; Animal: 0.4 mg/kg/day i.p. for 2 weeks	Model group (saline)	11. FOD ↓ IL-6/TNF-α.
**Cheng 2022[5]**	HDW aqueous extract	Mice: HDW 100 mg/kg/day orally for 8–12 weeks; Cell model: HDW extract 100–400 µg/mL for 24–48 h	HFD model group without HDW	1. HDW ↓ serum ALT/AST; 2. HDW ↓ hepatic TG; 3. HDW improved histology (reduced steatosis & lipid accumulation); 5. HDW ↓ inflammatory cytokines (TNF-α and IL-6); 6. HDW ↓ oxidative stress (ROS↓, MDA↓); 7. HDW ↑ SIRT1 and ↓ NF-κB p65 & ↓ p53 activation.
**Feng 2017[8]**	EEHDW	In vitro: 0–2 mg/mL for 24–48 h; In vivo: 1 g/kg/day oral gavage for 16 days	Vehicle control	7. ↓ COX-2, iNOS, eNOS, HIF-1α; 8. ↓ IL-1β, IL-6, TNF-α and ↑ IL-4, IL-10; 9. ↓ p-AKT, p-Erk1/2, p-JNK, p-p38, p-p70S6K, p-STAT3 and ↑ p-p53.
**Feng 2025[9]**	TFHDW (Total Flavonoids of HDW)	Cell lines: 10–160 µM (48 h); Functional assays at 40 µM (RM1) and 30 µM (LNCaP); Mice: 50 mg/kg/day oral gavage × 3 weeks	Blank/vehicle; si-PIAS4; oe-STAT3; MG-132; sh-PIAS4	6. ↑ PIAS4; 7. ↓ STAT3 activity; 8. STAT3 overexpression partially rescued phenotype.
**Han 2023[10]**	EEOD (Ethanol Extract of Oldenlandia diffusa)	2D assays: 0–8 mg/mL for 17–24 h; 3D invasion assay: 0, 1, 2, 3 mg/mL continuously perfused (1 µL/min) for 24 h	Control (0 mg/mL EEOD); Positive control: temozolomide 100 µmol/L	3. EEOD ↓ scratch migration in U87 (1–2 mg/mL) and U251 (2 mg/mL); 4. EEOD ↓ Transwell migration (dose-dependent); 5. EEOD ↓ invasion in Transwell and 3D spheroids (1–3 mg/mL); 8. EEOD suppresses glioma invasion in both 2D and 3D models.
**Huang 2022[13]**	Hedyotis diffusa Injection (HDI)	In vitro: 0–100 µg/mL, 24–48h; In vivo: 15 mg/kg/day IM × 21d	NS control, cisplatin	4. HDI caused mitochondrial shrinkage (ferroptosis morphology); 5. HDI ↑ lipid ROS; 6. HDI ↑ Fe^{2+} accumulation; 7. HDI ↑ MDA; 8. HDI ↓ Bcl-2, ↑ Bax, ↑ VDAC2/3, ↑ HMOX1/TFR (ferroptosis markers); 9. Blocking

				VDAC2/3 or GPX4 pathways reduced HDI-induced ferroptosis; 12. IHC: Bax↑, Bcl-2↓, VDAC2/3↑, 4-HNE↑, TFR↑, HMOX1↑.
**Jin 2022[54]**	2-hydroxy-3-methylanthraquinone (HMA)	50 μmol/L for 48 h	Vehicle (0 μmol/L)	1. HMA weakly ↑ apoptosis; 2. HMA ↓ migration; 3. HMA ↓ invasion; 4. HMA regulated 285 proteins (98 ↑, 187 ↓); 5. HMA suppressed cancer-related pathways including PI3K–Akt, Wnt, HIF-1, focal adhesion, microRNA pathways; 6. HMA ↓ IL-8 and ↓ ECM/motility-associated proteins, indicating TME modulation
**Jing 2023[15]**	2-hydroxy-3-methylanthraquinone (HMA)	0–200 μmol/L for 24–48 h (in vitro); 100 μmol/L HMA (in vivo, duration NR)	Vehicle control	7. RNA-seq: 3815 DEGs including ↓MYC; 8. HMA ↓ MYC mRNA/protein; 9. HMA ↓ CHK1 and ↓ RAD51 phosphorylation (T309) (DNA damage-response and repair pathways relevant to treatment sensitivity).
**Kim 2018[16]**	Ursolic acid (UA)	0–80 μM for 24 h	Vehicle control (0 μM)	5. UA ↓ p-JAK2 and ↓ p-STAT3; 6. UA blocked STAT3 nuclear translocation; 7. UA ↑ miR-4500 expression; 8. miR-4500 inhibitor reversed UA-induced cytotoxicity ↓, colony suppression reversed, apoptosis ↓, and p-STAT3 inhibition rescued, confirming miR-4500 → STAT3 axis involvement.
**Lai 2017[18]**	HDW ethanol extract (EEHDW)	0–2.0 mg/mL EEHDW for 12–48 h	Vehicle control	2. EEHDW ↓ migration (wound healing); 3. EEHDW ↓ migration & ↓ invasion (Transwell); 4. EEHDW ↓ adhesion capacity; 5. EEHDW ↓ TGF-β & ↓ SMAD4 & ↓ N-cadherin mRNA, ↑ E-cadherin mRNA; 6. EEHDW ↓ TGF-β & ↓ SMAD4 & ↓ N-cadherin protein, ↑ E-cadherin protein, indicating suppression of TGF-β/SMAD4-mediated EMT and metastasis inhibition.
**Lee 2019[20]**	Intracellular extract of OD	0–400 μg/mL for 24–48 h (in vitro); OD administered for 4 weeks (dose NR)	Vehicle control	4. OD ↑ E-cadherin and ↓ N-cadherin/vimentin, indicating EMT suppression; 5. OD ↑ AMPK activation (p-AMPK↑).
**Li 2019[55]**	EEHDW	CRC cells: 0–2.0 mg/mL for 24 h; HLECs: 0–0.5 mg/mL ± VEGF-C (5 ng/mL) for 24 h	Vehicle control (DMSO) ± VEGF-C only group	1. EEHDW ↓ CRC viability dose-dependently; 2. EEHDW ↓ CRC colony formation; 3. EEHDW ↓ CRC migration; 4. EEHDW ↓ VEGF-C expression & secretion; 5. EEHDW ↓ VEGF-C–induced HLEC viability; 6. EEHDW ↓ HLEC colony formation; 7. EEHDW had no significant effect on HLEC cell cycle; 8. EEHDW did not induce apoptosis in HLECs; 9. EEHDW ↓ VEGF-C–enhanced HLEC migration; 10. EEHDW ↓ VEGF-C–enhanced tube formation; 11. EEHDW ↓ MMP2, MMP9, cyclin D1, CDK4; 12. EEHDW ↓ VEGFR3 and ↓ PI3K/AKT, ↓ ERK, ↓ STAT3 activation, indicating strong anti-lymphangiogenesis activity
**Lin 2019a[56]**	Hedyotis diffusa polysaccharide (HDP)	25, 50, 100 μg/mL for 24 h	Vehicle control	1. HDP ↓ adhesion; 2. HDP ↓ migration; 3. HDP ↓ invasion; 4. HDP ↓ MMP-2/-9 enzymatic activity; 5. HDP ↓ MMP-2/-9 protein; 6. HDP ↑ TIMP-1/-2 activity; 7. HDP ↑ TIMP-1/-2 protein; 8. HDP ↑ E-cadherin and ↓ N-cadherin/vimentin (EMT inhibition); 9. HDP ↓ COX-2; 10. HDP ↓ p-EGFR; 11. HDP ↓ p-Akt; 12. HDP ↓ p-ERK1/2, with no major change in JNK/p38, indicating EMT and metastasis suppression through EGFR/Akt/ERK pathway inactivation

**Lv 2023[57]**	Hedyotis diffusa extract + Co(II) coordination polymers	CPs: NR μM; HDW extract: NR (dose not specified)	Vehicle control; CPs alone	1. HDW + CPs ↓ migration more strongly than CPs alone; 2. HDW + CPs ↓ invasion synergistically; 3. HDW + CPs ↑ E-cadherin and ↓ N-cadherin/vimentin mRNA levels, indicating cooperative EMT inhibition
**Ma 2019[58]**	Hedyotis diffusa polysaccharides (HDP)	In vitro: 10, 50, 100 μg/mL; In vivo: 10 mg/kg/day i.p.	HDP-free CIK (control); saline; CIK alone	1. CD3+CD56+↑ dose-dependent; 2. TNF-α+↑ and IFN-γ+↑; 3. Apoptosis↓; 4. Cytotoxicity↑ against all tumor lines; 5. CR3↑ and CR3 blockade ↓HDP-mediated activation; 6. In vivo tumor volume↓ and weight↓ greatest in CIK+HDP group
**Wang 2018b[59]**	Hedyotis diffusa decoction	3.0 g/kg orally once daily for 14 days	Model group (tumor-bearing, water gavage)	1. HDW partially restored urine metabolites including ornithine↓, N-acetyl-L-aspartate↑, L-aspartate↑, creatinine normalization; 2. HDW partially reversed plasma metabolic disturbances including acetate↑, lactate↓, choline↓, L-glutamine↓, 3-hydroxybutyrate↓; 3. HDW improved multiple tumor-associated pathways (amino acid metabolism, glycolysis, lipid metabolism) indicating metabolic rebalancing in tumor-bearing rats.
**Wang 2021b[36]**	OD extract ± isolated UA	In vitro doses: NR; In vivo dose: NR	Vehicle control	3. UA ↓ glycolysis (↓ glucose uptake, ↓ lactate); 4. UA ↑ Cav-1 via SP1 activation; 5. CAV1/SP1 silencing reversed anti-glycolytic and anti-proliferative effects.
**Wang 2023[38]**	Kaempferol treatment	0–80 μM for 24–48 h in vitro; 20 or 60 mg/kg/day i.p. in vivo for tumor-bearing nude mice	Vehicle control; Cisplatin (positive control in vitro/in vivo)	6. Kaempferol ↓ MET and p-MET mRNA/protein; 7. Kaempferol ↓ PI3K/p-PI3K, AKT/p-AKT, mTOR/p-mTOR (signaling axes involved in growth and microenvironmental regulation).
**Wu 2023[42]**	HDW injection	50, 100, 150 mg/kg i.p., every other day for 14 injections	Vehicle control	2. CD31↓ and α-SMA↓ indicating angiogenesis inhibition; 3. Liver index↓ vs vehicle; 4. IL-6↓, IL-1β↓, IL-17↓, TNF-α↓; 5. p-Akt1/Akt1↓, p-mTOR/mTOR↓, p-STAT3/STAT3↓, HIF-1α↓; 6. IHC confirmed HIF-1α↓, indicating antiangiogenesis via inflammatory and Akt/mTOR/STAT3/HIF-1α pathways.
**Yang 2019[44]**	Ethanol extract of Oldenlandia diffusa (EEOD)	20–50 μg/mL for 24–48 h (in vitro); 20–40 μg/mL in water (zebrafish model)	Vehicle control	6. ↓ MMP2, MMP9, N-cadherin, Vimentin, CAV1; 7. ↓ metastatic spread in zebrafish.
**Yang 2025[45]**	HDW 20:1 concentrated extract	2–6 mg/mL for 24–48 h (in vitro); 600 mg/kg by gavage daily × 21 days (in vivo)	Vehicle control (0 mg/mL or saline)	6. ↓ IL-17A and ↓ p-NF-κB p65 indicating pathway inhibition.
**Yao 2025[46]**	Fisetin extracted from HDW	50–300 μg/mL for 24 h	Vehicle control (culture medium or PBS)	4. PI3K↓, MTOR↓, HIF1A↓, VEGFA↓ proteins; PTEN↑; 5. HIF1A mRNA↓, VEGFA mRNA↓, PTEN mRNA↑; 6. Hemolysis <5%, indicating good biocompatibility (relevant to systemic/vascular compatibility).
**Zheng 2025[49]**	Quercetin from Hedyotis diffusa	Multiple doses; dose-dependent effects reported	Vehicle control (untreated)	3. Quercetin ↓ IL6 and TNF mRNA expression; 4. Quercetin ↓ IL6 and TNF protein levels; 5. Quercetin ↑ AGEs and RAGE protein levels, supporting activation of AGE-RAGE signaling as part of its anticancer mechanism.

**Zhou 2025[50]**	Hedyotis diffusa Willd. granules (water extract)	In vitro: 0–3 mg/mL for 0.5–48 h; In vivo: 150 or 300 mg/kg/day orally for 21 days	Vehicle control; FAK inhibitor (Y15 10 μM); Src inhibitor (PP2 10 μM)	4. HDW ↓ p-FAK and ↓ p-Src; Y15/PP2 attenuated HDW anti-adhesive effects indicating Src/FAK dependence; 6. HDW ↓ in vivo TC–platelet aggregates and TC–EC adhesion and ↓ p-FAK/p-Src in metastases.
**Zhu 2025[52]**	Active compounds of HDW (mix)	UA, OA, quercetin 0–70 μM for 72 h for cytotoxicity in SW480 cells; UA 12 μM for 72 h for TMT-based proteomics in SW480 cells; UA 10–20 μM for 24–72 h for RPLP1 and functional assays	DMSO vehicle control; shCtrl/NC for shRNA groups; UA alone vs RPLP1 knockdown alone vs combination	7. UA significantly ↓ RPLP1 mRNA ($p<0.01$–0.001); 8. TMT proteomics identified 804 DEPs (438↑, 366↓) enriched in complement/coagulation pathways, cell adhesion molecules, endopeptidase regulation and steroid biosynthesis; combined UA + RPLP1 knockdown synergistically ↓ proliferation, migration, invasion and ↑ apoptosis vs either alone.

AMPK, AMP-activated protein kinase; DC, dendritic cell; ECM, extracellular matrix; EGFR, epidermal growth factor receptor; EMT, epithelial–mesenchymal transition; FAK, focal adhesion kinase; GZMB, granzyme B; HDI, Hedyotis diffusa injection; HDP, Hedyotis diffusa polysaccharide; HDW, Hedyotis diffusa Willd.; HIF-1α, hypoxia-inducible factor-1α; IFN, interferon; IL, interleukin; MMP/TIMP, matrix metalloproteinase and tissue inhibitor; NF-κB, nuclear factor kappa B; NK, natural killer cell; PI3K/Akt/mTOR, phosphoinositide 3-kinase/Akt/mechanistic target of rapamycin pathway; ROS, reactive oxygen species; STAT3, signal transducer and activator of transcription 3; TME, tumor microenvironment; TNF, tumor necrosis factor; Treg, regulatory T cell; VEGF/VEGFR, vascular endothelial growth factor and receptor.

References

1. Bai, K.; Long, Y.; Yuan, F.; Huang, X.; Liu, P.; Hou, Y.; Zou, X.; Jiang, T.; Sun, J. Hedyotis diffusa injection modulates the ferroptosis in bladder cancer via CAV1/JUN/VEGFA. *Int Immunopharmacol* **2025**, *147*, 113925, doi:10.1016/j.intimp.2024.113925.
2. Chen, W.; Jin, Y.; Yang, H.; Wei, L.; Lin, J. Hedyotis diffusa Willd reduces migration and invasion through inhibition of TGF-β-induced EMT in colorectal cancer cells. *European Journal of Integrative Medicine* **2018**, *23*, 57–63, doi:10.1016/j.eujim.2018.09.008.
3. Chen, H.; Shang, X.; Yuan, H.; Niu, Q.; Chen, J.; Luo, S.; Li, W.; Li, X. Total flavonoids of Oldenlandia diffusa (Willd.) Roxb. suppresses the growth of hepatocellular carcinoma through endoplasmic reticulum stress-mediated autophagy and apoptosis. *Front Pharmacol* **2022**, *13*, 1019670, doi:10.3389/fphar.2022.1019670.
4. Chen, Q.; Xu, Y.; Ma, J.; Zhang, G.; Yin, Z.; Zhang, D.; Luo, D.; Liu, Z. Network Pharmacology Integrated Experimental Validation Uncover Quercetin as the Key Ingredient of Hedyotis diffusa Anti-BRCA. *Chem Biol Drug Des* **2025**, *105*, e70112, doi:10.1111/cbdd.70112.
5. Cheng, Y.Y.; Tuzo, E.T.; Dalley, J.W.; Tsai, T.H. Dose-dependent effects of Hedyotis diffusa extract on the pharmacokinetics of tamoxifen, 4-hydroxytamoxifen, and N-desmethyltamoxifen. *Biomed Pharmacother* **2022**, *145*, 112466, doi:10.1016/j.biopha.2021.112466.
6. Cheng, Q.; Zhang, D.; Zhao, X.; Zhu, L.; Tao, J.; Xiao, J.; Wang, R.; Leng, J. Composition analysis of extracellular vesicle-like particles derived from Hedyotis diffusa Willd and their effects on apoptosis in hepatocellular carcinoma Huh-7 cells. *Int J Biol Macromol* **2025**, *321*, 146395, doi:10.1016/j.ijbiomac.2025.146395.
7. Chung, T.W.; Choi, H.; Lee, J.M.; Ha, S.H.; Kwak, C.H.; Abekura, F.; Park, J.Y.; Chang, Y.C.; Ha, K.T.; Cho, S.H.; et al. Oldenlandia diffusa suppresses metastatic potential through inhibiting matrix metalloproteinase-9 and intercellular adhesion molecule-1 expression via p38 and ERK1/2 MAPK pathways and induces apoptosis in human breast cancer MCF-7 cells. *J Ethnopharmacol* **2017**, *195*, 309–317, doi:10.1016/j.jep.2016.11.036.
8. Feng, J.; Jin, Y.; Peng, J.; Wei, L.; Cai, Q.; Yan, Z.; Lai, Z.; Lin, J. Hedyotis diffusa willd extract suppresses colorectal cancer growth through multiple cellular pathways. *Oncol Lett* **2017**, *14*, 8197–8205, doi:10.3892/ol.2017.7244.
9. Feng, R.; Li, Z.; Jia, Y.; Ji, Y.; Guo, M.; Wang, X. Total Flavonoids of Hedyotis Diffusa Willd Suppresses Prostate Cancer Progression by Promoting AR Ubiquitination and Degradation via the PIAS4/STAT3 Pathway. *Cell Biol Int* **2025**, *49*, 1475–1492, doi:10.1002/cbin.70070.
10. Han, C.H.; Ma, J.Y.; Zou, W.; Qu, J.L.; Du, Y.; Li, N.; Liu, Y.; Jin, G.; Leng, A.J.; Liu, J. 3D Microfluidic System for Evaluating Inhibitory Effect of Chinese Herbal Medicine Oldenlandia diffusa on Human Malignant Glioma Invasion Combined with Network Pharmacology Analysis. *Chinese Journal of Integrative Medicine* **2023**, *29*, 52–60, doi:10.1007/s11655-021-3726-1.
11. Ho, K.L.; Lew, A.P.; Ong, Y.C.; Lew, Y.Q.; Wong, Z.H. Antiproliferative, antimigratory, and anticlonogenic effects of Hedyotis diffusa, Panax ginseng, and their combination on colorectal cancer cell lines. *Journal of Herbs, Spices and Medicinal Plants* **2018**, *24*, 185–198, doi:10.1080/10496475.2018.1428919.
12. Huang, L.; Xu, H.; Wu, T.; Li, G. Hedyotis diffusa Willd. Suppresses Hepatocellular Carcinoma via Downregulating AKT/mTOR Pathways. *Evid Based Complement Alternat Med* **2021**, *2021*, 5210152, doi:10.1155/2021/5210152.
13. Huang, F.; Pang, J.; Xu, L.; Niu, W.; Zhang, Y.; Li, S.; Li, X. Hedyotis diffusa injection induces ferroptosis via the Bax/Bcl2/VDAC2/3 axis in lung adenocarcinoma. *Phytomedicine* **2022**, *104*, 154319, doi:10.1016/j.phymed.2022.154319.
14. Jiang, J.; Wang, B.; Li, J.; Ye, B.; Lin, S.; Qian, W.; Shan, L.; Efferth, T. Total coumarins of Hedyotis diffusa induces apoptosis of myelodysplastic syndrome SKM-1 cells by activation of caspases and inhibition of PI3K/Akt pathway proteins. *J Ethnopharmacol* **2017**, *196*, 253–260, doi:10.1016/j.jep.2016.12.012.

15. Jing, D.; Chen, X.; Zhang, Z.; Chen, F.; Huang, F.; Zhang, Z.; Wu, W.; Shao, Z.; Pu, F. 2-Hydroxy-3-methylanthraquinone inhibits homologous recombination repair in osteosarcoma through the MYC-CHK1-RAD51 axis. *Mol Med* **2023**, *29*, 15, doi:10.1186/s10020-023-00611-y.
16. Kim, K.; Shin, E.A.; Jung, J.H.; Park, J.E.; Kim, D.S.; Shim, B.S.; Kim, S.H. Ursolic Acid Induces Apoptosis in Colorectal Cancer Cells Partially via Upregulation of MicroRNA-4500 and Inhibition of JAK2/STAT3 Phosphorylation. *Int J Mol Sci* **2018**, *20*, doi:10.3390/ijms20010114.
17. Kuo, Y.J.; Liu, Y.J.; Way, T.D.; Chiang, S.Y.; Lin, J.G.; Chung, J.G. Synergistic inhibition of leukemia WEHI-3 cell growth by arsenic trioxide and Hedyotis diffusa willd extract in vitro and in vivo. *Experimental and Therapeutic Medicine* **2017**, *13*, 3388–3396, doi:10.3892/etm.2017.4392.
18. Lai, Z.; Yan, Z.; Chen, W.; Peng, J.; Feng, J.; Li, Q.; Jin, Y.; Lin, J. Hedyotis diffusa Willd suppresses metastasis in 5-fluorouracil-resistant colorectal cancer cells by regulating the TGF-β signaling pathway. *Mol Med Rep* **2017**, *16*, 7752–7758, doi:10.3892/mmr.2017.7500.
19. Lee, S.; Shim, J.H.; Gim, H.; Park, H.S.; Kim, B.J. Ethanol Extract of Oldenlandia diffusa - an Effective Chemotherapeutic for the Treatment of Colorectal Cancer in Humans: -Anti-Cancer Effects of Oldenlandia diffusa. *J Pharmacopuncture* **2016**, *19*, 51–58, doi:10.3831/kpi.2016.19.007.
20. Lee, Y.K.; Lim, J.; Yoon, S.Y.; Joo, J.C.; Park, S.J.; Park, Y.J. Promotion of Cell Death in Cisplatin-Resistant Ovarian Cancer Cells through KDM1B-DCLRE1B Modulation. *Int J Mol Sci* **2019**, *20*, doi:10.3390/ijms20102443.
21. Li, Y.L.; Zhang, J.; Min, D.; Hongyan, Z.; Lin, N.; Li, Q.S. Anticancer Effects of 1,3-Dihydroxy-2-Methylanthraquinone and the Ethyl Acetate Fraction of Hedyotis Diffusa Willd against HepG2 Carcinoma Cells Mediated via Apoptosis. *PLoS One* **2016**, *11*, e0151502, doi:10.1371/journal.pone.0151502.
22. Li, P.; Li, J.; Liu, Y.; Guo, Y.; Liu, Y.; Ren, Y. Quantitative and qualitative determination of gallic acid in Hedyotis diffusa willd. Extract and its anti-lung cancer NCI-H460 cell activity. *Biomedical Research (India)* **2017**, *28*, 5280–5284.
23. Li, Q.; Lai, Z.; Yan, Z.; Peng, J.; Jin, Y.; Wei, L.; Lin, J. Hedyotis diffusa Willd inhibits proliferation and induces apoptosis of 5-FU resistant colorectal cancer cells by regulating the PI3K/AKT signaling pathway. *Mol Med Rep* **2018**, *17*, 358–365, doi:10.3892/mmr.2017.7903.
24. Lin, L.; Cheng, K.; Xie, Z.; Chen, C.; Chen, L.; Huang, Y.; Liang, Z. Purification and characterization a polysaccharide from Hedyotis diffusa and its apoptosis inducing activity toward human lung cancer cell line A549. *Int J Biol Macromol* **2019**, *122*, 64–71, doi:10.1016/j.ijbiomac.2018.10.077.
25. Ling, J.Y.; Wang, Q.L.; Liang, H.N.; Liu, Q.B.; Yin, D.H.; Lin, L. Flavonoid-Rich Extract of Oldenlandia diffusa (Willd.) Roxb. Inhibits Gastric Cancer by Activation of Caspase-Dependent Mitochondrial Apoptosis. *Chin J Integr Med* **2023**, *29*, 213–223, doi:10.1007/s11655-022-3679-4.
26. Lu, P.H.; Chen, M.B.; Ji, C.; Li, W.T.; Wei, M.X.; Wu, M.H. Aqueous Oldenlandia diffusa extracts inhibits colorectal cancer cells via activating AMP-activated protein kinase signalings. *Oncotarget* **2016**, *7*, 45889–45900, doi:10.18632/oncotarget.9969.
27. Lv, Y.; Wang, Y. Chemical constituents from Oldenlandia diffusa and their cytotoxic effects on human cancer cell lines. *Nat Prod Res* **2021**, *37*, 397–403, doi:10.1080/14786419.2021.1974434.
28. Ma, W.L.; Chang, N.; Yu, Y.; Su, Y.T.; Chen, G.Y.; Cheng, W.C.; Wu, Y.C.; Li, C.C.; Chang, W.C.; Yang, J.C. Ursolic acid silences CYP19A1/aromatase to suppress gastric cancer growth. *Cancer Med* **2022**, *11*, 2824–2835, doi:10.1002/cam4.4536.
29. Ning, W.; Xu, N.; Zhou, C.; Zou, L.; Quan, J.; Yang, H.; Lu, Z.; Cao, H.; Liu, J. Ethyl Acetate Fraction of Hedyotis diffusa Willd Induces Apoptosis via JNK/Nur77 Pathway in Hepatocellular Carcinoma Cells. *Evid Based Complement Alternat Med* **2022**, *2022*, 1932777,

doi:10.1155/2022/1932777.

30. Ou, L.; Li, M.; Hou, Y. Network pharmacology, bioinformatics, and experimental validation to identify the role of Hedyotis diffusa willd against gastric cancer through the activation of the endoplasmic reticulum stress. *Heliyon* **2024**, *10*, e28833, doi:10.1016/j.heliyon.2024.e28833.
31. Pu, F.; Chen, F.; Lin, S.; Chen, S.; Zhang, Z.; Wang, B.; Shao, Z. The synergistic anticancer effect of cisplatin combined with Oldenlandia diffusa in osteosarcoma MG-63 cell line in vitro. *Onco Targets Ther* **2016**, *9*, 255–263, doi:10.2147/ott.S90707.
32. Sun, G.; Wei, L.; Feng, J.; Lin, J.; Peng, J. Inhibitory effects of Hedyotis diffusa Willd. on colorectal cancer stem cells. *Oncol Lett* **2016**, *11*, 3875–3881, doi:10.3892/ol.2016.4431.
33. Trang, V.M.; Son, N.T.; Luyen, N.D.; Pham, T.V.; Giang, P.M.; Duong, P.A.; Huong, D.T.V. Hedyotis diffusa Aerial Parts: Essential Oil Composition, Biological Activity, and In Silico Approach. *Chem Biodivers* **2025**, *22*, e00010, doi:10.1002/cbdv.202500010.
34. Wang, C.; Zhou, X.; Wang, Y.; Wei, D.; Deng, C.; Xu, X.; Xin, P.; Sun, S. The Antitumor Constituents from Hedyotis Diffusa Willd. *Molecules* **2017**, *22*, doi:10.3390/molecules22122101.
35. Wang, C.; Xin, P.; Wang, Y.; Zhou, X.; Wei, D.; Deng, C.; Sun, S. Iridoids and sfingolipids from Hedyotis diffusa. *Fitoterapia* **2018**, *124*, 152–159, doi:10.1016/j.fitote.2017.11.004.
36. Wang, S.; Chang, X.; Zhang, J.; Li, J.; Wang, N.; Yang, B.; Pan, B.; Zheng, Y.; Wang, X.; Ou, H.; et al. Ursolic Acid Inhibits Breast Cancer Metastasis by Suppressing Glycolytic Metabolism via Activating SP1/Caveolin-1 Signaling. *Front Oncol* **2021**, *11*, 745584, doi:10.3389/fonc.2021.745584.
37. Wang, B.; Jiang, J.; Zhang, Y.; Shen, Y.; Wu, L.; Tang, S.; Lin, S. Combination of HDE and BIIB021 efficiently inhibits cell proliferation and induces apoptosis via downregulating hTERT in myelodysplastic syndromes. *Exp Ther Med* **2021**, *21*, 503, doi:10.3892/etm.2021.9934.
38. Wang, R.; Deng, Z.; Zhu, Z.; Wang, J.; Yang, X.; Xu, M.; Wang, X.; Tang, Q.; Zhou, Q.; Wan, X.; et al. Kaempferol promotes non-small cell lung cancer cell autophagy via restricting Met pathway. *Phytomedicine* **2023**, *121*, 155090, doi:10.1016/j.phymed.2023.155090.
39. Wang, S.; Yin, N.; Li, Y.; Ma, Z.; Lin, W.; Zhang, L.; Cui, Y.; Xia, J.; Geng, L. Molecular mechanism of the treatment of lung adenocarcinoma by Hedyotis Diffusa: an integrative study with real-world clinical data and experimental validation. *Front Pharmacol* **2024**, *15*, 1355531, doi:10.3389/fphar.2024.1355531.
40. Wu, C.; Luo, H.; Ma, W.; Ren, X.; Lu, C.; Li, N.; Wang, Z. Polysaccharides isolated from Hedyotis diffusa inhibits the aggressive phenotypes of laryngeal squamous carcinoma cells via inhibition of Bcl-2, MMP-2, and μPA. *Gene* **2017**, *637*, 124–129, doi:10.1016/j.gene.2017.09.041.
41. Wu, K.; Wu, X.; Liang, Y.; Wang, T.; Wu, D.; Li, L.; Wang, Z. Inhibitory effects of total triterpenoids isolated from the Hedyotis diffusa willd on H1975 cells. *Front Pharmacol* **2022**, *13*, 922477, doi:10.3389/fphar.2022.922477.
42. Wu, H.; Zhang, L.; Wang, C.; Li, F.; Qi, L.; Xiao, L.; Zhang, M.; Zhang, H.; Zhang, G.; Qin, Y. Network Pharmacology Analysis and Experimental Verification on Antiangiogenesis Mechanism of Hedyotis diffusa Willd in Liver Cancer. *Evid Based Complement Alternat Med* **2023**, *2023*,

1416841, doi:10.1155/2023/1416841.
43. Yan, Z.; Feng, J.; Peng, J.; Lai, Z.; Zhang, L.; Jin, Y.; Yang, H.; Chen, W.; Lin, J. Chloroform extract of Hedyotis diffusa Willd inhibits viability of human colorectal cancer cells via suppression of AKT and ERK signaling pathways. *Oncol Lett* **2017**, *14*, 7923–7930, doi:10.3892/ol.2017.7245.
44. Yang, B.; Wang, N.; Wang, S.; Li, X.; Zheng, Y.; Li, M.; Song, J.; Zhang, F.; Mei, W.; Lin, Y.; et al. Network-pharmacology-based identification of caveolin-1 as a key target of Oldenlandia diffusa to suppress breast cancer metastasis. *Biomed Pharmacother* **2019**, *112*, 108607, doi:10.1016/j.biopha.2019.108607.
45. Yang, S.; Zeng, L.; Deng, C.; Tian, X.; Sun, W.; Ji, C.; Zhang, Q. Anti-tumor Effects of Hedyotis diffusa Willd on Cervical Cancer: Inhibition of Proliferation, Migration, and Induction of Apoptosis. *Iran J Pharm Res* **2025**, *24*, e159390, doi:10.5812/ijpr-159390.
46. Yao, H.; Wang, D.; Ye, J.; Cong, H.; Yu, B. Investigation on the antitumor effects of fisetin extracted from Hedyotis diffusea willd based on network pharmacology and experimental validation. *J Ethnopharmacol* **2025**, *353*, 120288, doi:10.1016/j.jep.2025.120288.
47. Yuan, X.; Huang, H.; Yu, C.; Tang, Z.; Li, Y. Network pharmacology and experimental verification study on the mechanism of Hedyotis diffusa Willd in treating colorectal cancer. *Naunyn Schmiedebergs Arch Pharmacol* **2024**, *397*, 6507–6521, doi:10.1007/s00210-024-03024-8.
48. Zhang, L.; Zhang, J.; Qi, B.; Jiang, G.; Liu, J.; Zhang, P.; Ma, Y.; Li, W. The anti-tumor effect and bioactive phytochemicals of Hedyotis diffusa willd on ovarian cancer cells. *J Ethnopharmacol* **2016**, *192*, 132–139, doi:10.1016/j.jep.2016.07.027.
49. Zheng, Q.; Wu, X.; Peng, S. The immunotherapy mechanism of Hedyotis Diffusae Herba in treating liver cancer: a study based on network pharmacology, bioinformatics, and experimental validation. *Naunyn Schmiedebergs Arch Pharmacol* **2025**, *398*, 951–965, doi:10.1007/s00210-024-03312-3.
50. Zhou, S.; Dong, Q.; Li, X.; Jiao, Y.; Deng, X.; Guo, Z.; Hao, H.; Han, S. Hedyotis Diffusa Willd. Inhibits Pulmonary Metastasis of Cancer Through Suppression Of Src/Fak Signaling. **2025**, doi:10.2139/ssrn.5316240.
51. Zhu, H.; Zheng, Z.; Zhang, J.; Liu, X.; Liu, Y.; Yang, W.; Liu, Y.; Zhang, T.; Zhao, Y.; Liu, Y.; et al. Anticancer effect of 2,7-dihydroxy-3-methylanthraquinone on human gastric cancer SGC-7901 cells in vitro and in vivo. *Pharm Biol* **2016**, *54*, 285–292, doi:10.3109/13880209.2015.1033563.
52. Zhu, L.M.; Shi, H.X.; Xu, Z.Y.; Deng, H.B. TMT-based proteomics analysis identifies RPLP1 as a key protein target in ursolic acid Inhibition of colorectal cancer. *Discov Oncol* **2025**, *16*, 1665, doi:10.1007/s12672-025-03486-z.
53. Bai, L.; Liu, X.; Yuan, Z.; Xu, G.; Li, X.; Wan, Z.; Zhu, M.; Liang, X.; Li, P.; Lan, Q.; et al. Activation of IL-2/IL-2R pathway by Hedyotis diffusa polysaccharide improves immunotherapy in colorectal cancer. *Int J Biol Macromol* **2025**, *306*, 141013, doi:10.1016/j.ijbiomac.2025.141013.
54. Jin, H.; Cui, M. Recognition of potential therapeutic role of 2-hydroxy-3-methylanthraquinones in the treatment of gallbladder carcinoma: A proteomics analysis. *Fundam Clin Pharmacol* **2022**, *36*, 350–362, doi:10.1111/fcp.12740.

55. Li, H.; Lai, Z.; Yang, H.; Peng, J.; Chen, Y.; Lin, J. Hedyotis diffusa Willd. inhibits VEGF-C-mediated lymphangiogenesis in colorectal cancer via multiple signaling pathways. *Oncol Rep* **2019**, *42*, 1225–1236, doi:10.3892/or.2019.7223.
56. Lin, L.; Cheng, K.; He, Z.; Lin, Q.; Huang, Y.; Chen, C.; Xie, Z.; Chen, L.; Liang, Z. A polysaccharide from Hedyotis diffusa interrupts metastatic potential of lung adenocarcinoma A549 cells by inhibiting EMT via EGFR/Akt/ERK signaling pathways. *Int J Biol Macromol* **2019**, *129*, 706–714, doi:10.1016/j.ijbiomac.2019.02.040.
57. Lv, X.; Zhou, L.; Xu, L. Synergistic Effect of Co(II) Coordination Polymers with Hedyotis diffusa on Lung Cancer through Regulating the Expression of EMT Protein. *Latin American Journal of Pharmacy* **2023**, *42*, 356–362.
58. Ma, C.; Wei, Y.; Liu, Q.; Xin, Y.; Cao, G.; Wang, X.; Yang, P. Polysaccharides from Hedyotis diffusa enhance the antitumor activities of cytokine-induced killer cells. *Biomed Pharmacother* **2019**, *117*, 109167, doi:10.1016/j.biopha.2019.109167.
59. Wang, Z.; Gao, K.; Xu, C.; Gao, J.; Yan, Y.; Wang, Y.; Li, Z.; Chen, J. Metabolic effects of Hedyotis diffusa on rats bearing Walker 256 tumor revealed by NMR-based metabolomics. *Magn Reson Chem* **2018**, *56*, 5–17, doi:10.1002/mrc.4658.
